# Phosphorus availability mediates pathway-specific nitrogen cycling in stratified peatland microbiomes

**DOI:** 10.1093/ismeco/ycag143

**Published:** 2026-05-23

**Authors:** Shuaizhi Guo, Niall P McNamara, Gary D Bending, Ryan M Mushinski

**Affiliations:** School of Life Sciences, Gibbet Hill Campus, University of Warwick, Coventry CV4 7AL, West Mindlands, United Kingdom; UK Centre for Ecology & Hydrology, Lancaster Environment Centre, Library Avenue, Lancaster LA1 4AP, Lancashire, United Kingdom; School of Life Sciences, Gibbet Hill Campus, University of Warwick, Coventry CV4 7AL, West Mindlands, United Kingdom; School of Life Sciences, Gibbet Hill Campus, University of Warwick, Coventry CV4 7AL, West Mindlands, United Kingdom

**Keywords:** nitrogen cycling, phosphorus limitation, peatlands, metagenomics, denitrification, nitrification, blanket bog

## Abstract

Peatland microbiomes regulate nitrogen (N) cycling processes that control nutrient retention and greenhouse gas emissions in carbon-rich ecosystems. Although depth-driven redox gradients structure microbial communities, how physicochemical stratification shapes the functional versus taxonomic organization of N-cycling microorganisms remains unclear. We used shotgun metagenomics to characterize N-cycling gene distributions, taxonomic affiliations, and metagenome-assembled genomes (MAGs) across depth and vegetation gradients in a temperate blanket bog. Depth emerged as the primary structuring factor, creating functional-taxonomic decoupling. Surface peat (0–20 cm) harbored functionally diverse but taxonomically constrained communities assembled deterministically around nitrification and labile N acquisition, while subsurface peat (20–40 cm) supported taxonomically richer but functionally-simpler communities assembled stochastically and enriched in denitrification and dissimilatory nitrate reduction. Linear mixed-effects models revealed pathway-specific controls on N cycling. Denitrification increased with depth (β = 11.53, *P* < .05), whereas organic N transformation declined (β = −5.81, *P* < .05); depth effects on nitrification and N fixation became non-significant after accounting for environmental variables. Phosphorus (P) emerged as the strongest environmental predictor, regulating nitrification (β = 95.40, *P* < .01), N fixation (β = 128.33, *P* < .01), organic N transformation (β = 80.53, *P* < .01), and denitrification (β = −109.63, *P* < .05), highlighting the importance of P availability in structuring microbial N cycling. This challenges traditional N-limitation paradigms in ombrotrophic systems. MAGs revealed Pseudomonadota as the dominant N-cycling lineage, while incomplete denitrification capacity indicated genetic potential for N_2_O accumulation in subsurface layers. These findings demonstrate that P availability, rather than N content alone, regulates microbial N transformation capacity in peatlands, with implications for predicting nutrient dynamics under altered hydrological and nutrient deposition regimes.

## Introduction

Peatland ecosystems store ~644 Gt of carbon (C) despite covering only 3% of the global terrestrial surface [1]. Blanket bogs, which develop in oceanic climates with consistently high precipitation and cool temperatures, are particularly important in the United Kingdom, where they comprise 87% of the 1.75 Gt C stored in UK peatlands [2]. These systems are regulated by hydrological processes and vegetation composition [3, 4], with water table position controlling the distribution of aerobic and anaerobic soil volumes [5]. Elevated water tables create oxygen-deficient conditions that slow decomposition and promote peat accumulation [1]. However, ~50% of UK peatlands have been drained [2], and climate change is projected to further lower water tables through reduced precipitation and enhanced evapotranspiration [6], potentially converting these ecosystems from C sinks to sources.

Blanket bogs are ombrotrophic ecosystems that have historically been considered nitrogen (N)-limited [7]. However, recent evidence challenges this paradigm. Multiple studies have demonstrated phosphorus (P) limitation or N:P co-limitation in both ombrotrophic and minerotrophic peatlands [8–10], indicating that P availability can regulate microbial N-cycling capacity independently of total N pools. Understanding the interactive effects of P and N availability on microbial processes remains a critical knowledge gap with implications for nutrient retention and greenhouse gas emissions.

Global peatlands store 5.9–25.9 Gt of N [11], equivalent to 8%–37% of the global soil N pool as originally estimated by Limpens *et al.* [12]. Reactive N enters these systems via atmospheric deposition (0.34–2.92 g N m^−2^ y^−1^), biological N fixation (0.1–2.5 g N m^−2^ y^−1^), and N mineralization (0.1–5.9 g N m^−2^ y^−1^), with combined inputs often exceeding typical plant demand of 2–3 g N m^−2^ y^−1^ [13–16]. Microbial communities regulate the fate of this reactive N through interconnected processes including nitrification, denitrification, dissimilatory nitrate reduction to ammonium (DNRA), and anaerobic ammonium oxidation (ANAMMOX) [17]. Denitrification contributes up to 66% of N removal as gaseous losses [18], highlighting the central role of microorganisms in N retention versus loss. Microbial N-cycling functions can be characterized through quantification of genes encoding key enzymatic steps. These include *nif, vnf*, and *anf* genes for N-fixation; *amo, hao*, and *nxr* genes for nitrification; *nar, nap, nir, nor*, and *nos* genes for denitrification; *nrf* genes for DNRA; and diverse genes for organic N transformation including urease and glutamate/glutamine metabolism [19–21].

The vertical stratification in these ecosystems creates distinct microbial habitats. The surface acrotelm, typically extending from 0 to 20 cm depth, experiences intermittent oxygen exposure associated with water table fluctuations and receives fresh organic matter inputs, supporting aerobic metabolism, and rapid decomposition [22–25]. Below this, the catotelm (>20 cm depth) is characterized by persistent anoxia and accumulation of recalcitrant organic compounds. This transition, which corresponds to water table fluctuations, represents a fundamental shift in redox conditions and resource availability. Concurrent vertical gradients in C, N, and P concentrations further shape stoichiometric constraints on microbial activity [26, 27]. While vegetation composition influences peatland biogeochemistry [28, 29], natural blanket bogs contain mixed plant assemblages with intertwined root systems [30], potentially dampening vegetation-specific effects relative to depth-driven environmental gradients.

Despite the collective biogeochemical understanding, fundamental questions remain, including how depth-driven physicochemical gradients shape the decoupling of functional gene diversity from taxonomic diversity, and does P availability regulate microbial N-cycling capacity as strongly as, or more than, total N content? To answer these questions, we investigated the following objectives. First, we determined how peat depth and vegetation composition affect N-cycling gene abundance and diversity. Second, we identified which physicochemical factors, particularly N and P stoichiometry, control the distribution of N-cycling genes. Third, we characterized the specific microorganisms mediating N transformations through reconstruction of metagenome-assembled genomes (MAGs), enabling linkages between taxonomic identity and functional potential across the peat profile.

## Materials and methods

### Study site description and sampling

The study was conducted at Moor House National Nature Reserve in the Northern Pennines, England (54°39′N, 2°45′W), an ombrotrophic blanket bog dominated by *Sphagnum* mosses, *Calluna vulgaris*, and *Eriophorum vaginatum* [31]. The site lies at 550 m elevation, with mean annual temperature of 5.8°C and mean annual precipitation of 2048 mm [32]. Between 2014 and 2024, water table depth fluctuated from above the surface to −20 cm (Fig. S1). Sampling was performed in July 2024 across three vegetation types (*Sphagnum, Calluna, Eriophorum*), with three replicate cores per type. This timing represents peak summer conditions when water table depth averaged −8 cm and microbial activity is typically most pronounced. Plots represented mixed vegetation communities where one species was largely dominant. Peat cores were extracted to 40 cm using a Russian peat corer and divided into surface (0–20 cm) and subsurface (20–40 cm) layers. The two depth intervals were selected to represent functionally distinct biogeochemical zones within the peat profile. The surface layer (0–20 cm) corresponds to the acrotelm, which receive fresh organic matter inputs and experience intermittent oxygen exposure associated with water table fluctuations. The subsurface layer (20–40 cm) captures the transition to the largely anoxic catotelm, where conditions are generally more reduced due to persistent water saturation and microbial decomposition is dominated by anaerobic processes. These depth intervals align with typical water table dynamics at Moor House (Fig. S1), providing a mechanistic basis for anticipated functional stratification. Peat cores were placed in sterile Nasco Whirl-Pak™ giant-size sample bags immediately after collection and stored in a cold box containing ice packs in the field. Samples were transported to the laboratory within the same day [33]. Upon arrival, peat from the defined depth intervals was sectioned and homogenized prior to subsampling. Subsamples designated for molecular analyses were transferred into 50 ml Falcon tubes and stored at −20°C, and deoxyribonucleic acid (DNA) extraction was performed within 7 days of sampling. Subsamples for physicochemical analyses were kept in the original sample bags at 4°C prior to processing and were also analysed within 7 days.

### Peat physicochemical properties

Peat pH was measured in 1:2.5 peat:water extracts. Moisture content was determined gravimetrically (60°C, 48 h). Total C, N, and S were quantified by elemental analyser (Elementar precisION, CNS mode), where 5 mg of oven-dried, ground peat was combusted in an oxygen-rich environment. Total P was determined following nitric-peroxide block digestion, with colorimetric determination using the Murphy-Riley method [34]. Raw physicochemical measurements corresponding to the sequenced samples are provided in Table S1, linked to their NCBI BioSample accession numbers. Differences between surface and subsurface peat layers are reported in Table S2.

### Deoxyribonucleic acid extraction and metagenomic analysis

DNA was extracted from 0.25 g peat using the DNeasy PowerSoil Pro Kit (Qiagen). DNA quality was assessed by fluorometry and spectrophotometry, sheared to ~500 bp, and used to construct indexed metagenomic libraries (ALFA-SEQ kit). A total of 18 metagenomes were generated and analysed in this study, including 9 surface and 9 subsurface peat samples. Sequencing was performed on an Illumina NovaSeq 6000 platform, generating 150 bp paired-end reads. Sequencing yielded 121.9 ± 17.6 million raw reads per sample, of which 77.3 ± 10.1 million high-quality reads remained after quality control with Fastp [35]. Each sample was processed with the SqueezeMeta pipeline (v1.6.3) [36]. Assembly was performed with MEGAHIT (v1.2.9) [37], retaining contigs ≥200 bp, and open reading frames were predicted using Prodigal (v2.6.3) [38]. Functional annotation was conducted by aligning predicted proteins against the KEGG database [39] using DIAMOND (v2.0.13) [40] and HMMER (v3.3) [41]. Taxonomic classification was based on homology searches against the NCBI-nr database [42] using DIAMOND, with assignments refined using a Last Common Ancestor algorithm. Taxonomic assignments derived from read-based metagenomic profiling were based on the NCBI-nr database. Family-level taxonomic resolution was selected for the downstream analyses as it provides sufficient phylogenetic depth while maintaining robustness against binning uncertainties.

Gene abundances were normalized for gene length and sequencing depth using transcripts per million (TPM), whereby read counts mapped to each gene were divided by gene length and then scaled by the sum of length-normalized counts across the full gene catalog, yielding compositional relative abundances (i.e. TPM-normalized proportions prior to scaling). Rarefied read counts were further used exclusively for diversity analyses, while compositional analyses were conducted using centered log-ratio transformed gene abundance and microbial phylum-level percentage compositions. The core N-cycling gene pool comprised seventy KEGG orthologs representing nine functional categories, including N fixation, ammonia oxidation, hydroxylamine oxidation/reduction, nitrite oxidation, nitroalkane oxidation, nitrate reduction, nitrite reduction to ammonium, nitric oxide (NO) reduction, and organic N transformation (Table S3). Nitrogen Cycling Database (NCycDB) was used to specifically distinguish ammonia monooxygenase (*amo*) and methane monooxygenase (*pmo*) genes [43]. Comparisons between KEGG and NCycDB annotations were subsequently performed to evaluate potential interference from *pmo* sequences and to assess the robustness of gene identification. Consistency between KEGG- and NCycDB-based gene annotations was assessed using both Spearman and Pearson correlation analyses. Spearman correlation was used to evaluate whether the rank order of gene abundances across samples was preserved, whereas Pearson correlation assessed whether the magnitude of abundance variation followed a similar linear relationship between annotation approaches. Main results are presented in Section 3.1 and detailed statistics provided in Tables S4, S5 and S6. P cycling genes were identified using the Phosphorus Cycling Database (PCycDB), a curated database of functional gene families involved in microbial P transformations [44]. Gene annotations were screened against PCycDB to identify genes associated with organic P mineralization, inorganic P solubilization, and P-starvation response regulation (PSRR) [45]. The specific genes included in each functional category are listed in Table S7.

MAGs were reconstructed by binning contigs with MetaBAT2 [46] and CONCOCT [47], followed by integration with DAS Tool [48]. MAG quality was assessed with CheckM (v1.1.3) [49]; bins with completeness >75% and contamination <5% were retained, yielding 140 high-quality MAGs. Taxonomic classification used GTDB-Tk (v2.3.2) [50]. This approach was adopted to ensure consistent phylogenomic placement and improved taxonomic resolution, particularly at lower ranks (e.g. genus level), where classifications based on NCBI-nr annotations can be incomplete for environmental genomes. Predicted ORFs were annotated against KEGG using DIAMOND (e-value ≤1e^−5^, ≥30% identity, ≥25 amino acids alignment). MAG abundance was estimated as TPM based on read mapping to reconstructed MAG contigs using CoverM [51]. The proportion of reads mapping to MAGs ranged from 2.1% to 18.4% across samples, reflecting variation in the fraction of the microbial community represented by recovered genomes rather than differences in sequencing depth. TPM normalization was therefore used to enable comparison of relative MAG abundances across samples.

### Statistical analysis

Data normality and homogeneity were evaluated using Shapiro–Wilk and Bartlett tests. As data did not satisfy normality, non-parametric approaches were applied. Differences in peat physicochemical properties, gene or pathway abundance, and alpha-diversity between layers were assessed using Wilcoxon rank-sum tests. *P*-values were adjusted using Benjamini–Hochberg correction (FDR < 0.05). For vegetation comparisons, Kruskal–Wallis tests were followed by pairwise Wilcoxon rank-sum tests with Bonferroni correction. Given limited replication (*n* = 3 per vegetation type per depth), vegetation comparisons are exploratory. Principal component analysis (PCA) assessed variation in N-cycling gene composition using z-score standardized KEGG ortholog abundances (prcomp function). Squared loadings quantified gene contributions, aggregated by functional category. Permutational multivariate analysis of variance tested compositional differences using adonis with Bray–Curtis dissimilarity. Normalized stochasticity ratio (NST) determined deterministic versus stochastic assembly processes (999 permutations). Co-occurrence networks were inferred using the Sparse Correlations for Compositional data (SparCC) algorithm [52], which accounts for the compositional nature of sequencing-based abundance data. SparCC correlations were calculated from the gene abundance matrix across samples, and statistical significance was assessed using permutation-based pseudo *P*-values generated from 100 bootstrap permutations. Only robust associations (|r| ≥ 0.3, *P* < .05) were retained for network construction. Network structure was assessed using standard topological metrics, including the number of nodes, number of edges, network density (the ratio of observed edges to the total number of possible edges), and average degree. Network topology was then processed in Cytoscape (v3.10.3) [53] and visualized in Gephi (v0.9.2) [54]. Linear mixed-effects models were used to assess relationships between environmental variables (depth, C, N, P, S, EC, pH, and moisture) and the CLR-transformed abundance of N-cycling gene categories, with core identity included as a random intercept to account for non-independence of paired samples within peat cores. For each environmental predictor, models were fitted separately for each N-cycling category (e.g. nitrification, denitrification, N fixation, DNRA, and organic N transformation), and the resulting coefficients were then compiled across categories for comparison. To avoid overfitting and multicollinearity arising from the limited number of independent cores and the strong covariation among environmental variables, predictors were evaluated individually rather than included simultaneously. Thus, each model took the form CLR-transformed abundance of a given category ~ Predictor + (1 | Core_ID). In addition, a depth-adjusted model including depth and major nutrient variables (CLR-transformed abundance ~ Depth + C + N + P + S + (1 | Core_ID)) was used to assess whether observed depth effects were independent of underlying environmental gradients. For visualization, regression coefficients were standardized by z-scaling (mean = 0, SD = 1) within each pathway. Mantel tests determined correlations of physicochemical properties with gene composition. Stratification was quantified via log_2_(Surface/Subsurface) ratios. Spearman correlations assessed variable relationships. All statistical analyses were conducted in R (v4.4.1) within the RStudio environment. Data processing and manipulation were performed using the tidyverse suite of packages, including dplyr, tidyr, and stringr. Ecological and statistical analyses were conducted using the vegan package. Compositional data transformations were performed using the zCompositions, while the following correlation network analyses and visualization were implemented using linkET. Figures were generated using ggplot2, with improved label placement implemented using ggrepel, and color palettes were applied using RColorBrewer.

## Results

### Depth-structured diversity of nitrogen-cycling genes in peat microbiomes

**Figure 1:**
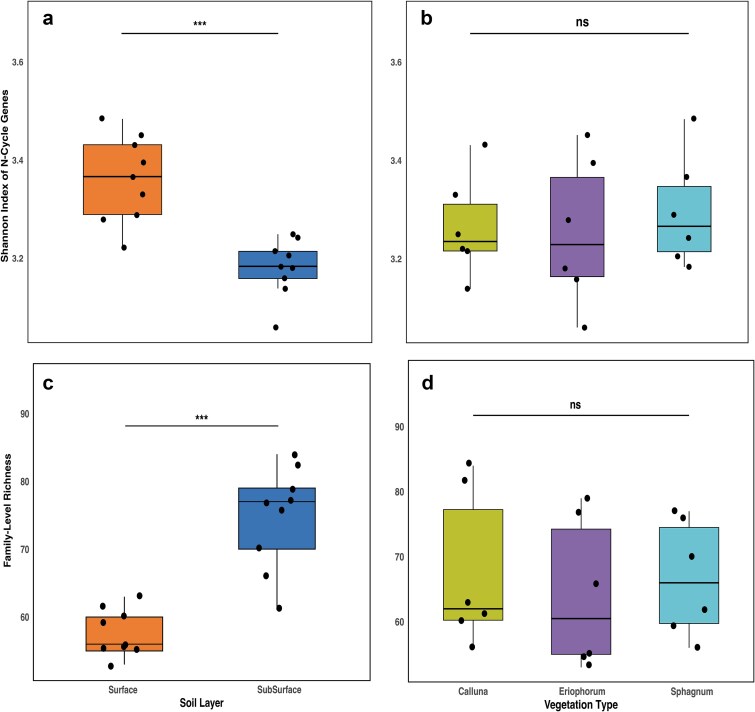
Alpha diversity patterns of N-cycling genes and microbial taxonomic richness across peat bog depths and vegetation types. (a and b) Shannon diversity index of 70 N-cycling genes compared between subsurface and surface soil layers (a) and across vegetation types (b). (c and d) family-level taxonomic richness compared between soil layers (c) and vegetation types (d). Boxplots show median, quartiles, and individual data points. Asterisks denote statistically significant differences (^***^P < .001; ns = not significant).

In this study, a total of 70 N-cycling genes were identified through annotation. Depth-dependent stratification was pronounced across all functional categories (Fig. 1). To evaluate whether sequence homology between *amo* and *pmo* genes could affect interpretation of nitrification patterns, KEGG-based annotations were validated against NCycDB, which explicitly distinguishes *amo* and *pmo* gene families. Across all 18 samples, KEGG and NCycDB annotations showed strong concordance for *amoA* abundance (Spearman ρ = 0.940, *P* < .001; Pearson r = 0.967, *P* < .001), and other key marker genes and derived functional ratios used in downstream interpretation (*norB, nosZ, ureC*; *amoA/nosZ, amoA/norB*, and *amoA/ureC*) were also significantly correlated between databases (Spearman ρ = 0.639–0.959; Pearson r = 0.612–0.978; Tables S4–S6). KEGG *amo* genes were also highly correlated with NCycDB *pmo* genes (Spearman ρ = 0.918–0.974; Table S4), consistent with the known sequence homology between these enzyme systems. Importantly, despite this overlap, the relative ecological patterns across samples remained highly consistent between annotation approaches. Because NCycDB recovered fewer N-cycling genes overall than KEGG (55 vs. 70) and did not identify *amoB* or *amoC* as *amo* genes in our dataset, KEGG-based annotations were retained for the main analyses.

**Figure 2:**
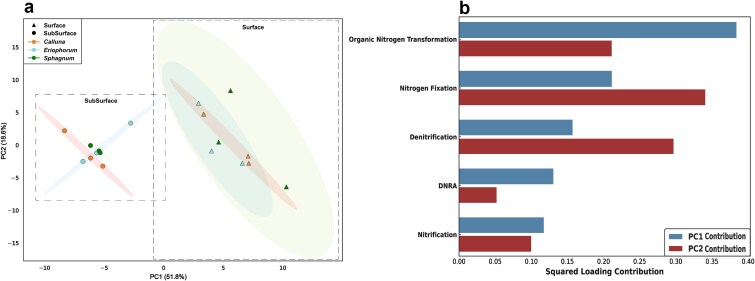
PCA of N-cycling gene composition and functional pathway contributions. (a) PCA ordination of samples based on standardized KEGG ortholog abundances of N-cycling genes. Points represent individual samples colored by vegetation type (orange = *Calluna*, blue = *Eriophorum*, green = *Sphagnum*) and shaped by soil depth (triangles = surface, circles = subsurface). Shaded ellipses represent 95% confidence intervals for each depth layer. PC1 and PC2 explain 51.8% and 18.6% of total variance, respectively. (b) Functional pathway contributions to principal components based on squared PCA loadings. Horizontal bars show the relative contribution of each N-cycling pathway to PC1 (blue) and PC2 (red), with pathways ordered by decreasing total contribution across both axes.

Shannon diversity of functional genes was significantly higher in surface than subsurface peat (*P* < .001), indicating greater functional diversity in the upper zone (Fig. 1a). Surface samples showed nitrification genes comprising 51% of the N-cycling gene pool compared to 28% in subsurface layers (*P* < .001), with ammonia oxidation genes alone representing 30% of surface functional profiles but only 6% in subsurface (*P* < .001). Conversely, denitrification genes were enriched in subsurface peat (32% vs. 17.8% in surface; *P* < .001), while DNRA-associated genes showed the strongest depth differentiation (4% subsurface vs. 0.4% surface; *P* < .001). N fixation genes also showed surface enrichment (28% vs. 21%; *P* < .001). Taxonomic richness at the family level exhibited the opposite pattern, with ~75 unique families in subsurface samples compared to 57 in surface layers (*P* < .001; Fig. 1c), revealing functional-taxonomic decoupling across depth. However, family-level Shannon diversity did not differ significantly between depths (Fig. S2a), as the strength of the depth effect varies slightly depending on the diversity metric used. In contrast, both richness and Shannon diversity at the order and genus levels showed significantly higher diversity in subsurface communities compared with surface communities (Fig. S2b and c), supporting the conclusion that subsurface microbial communities generally exhibit higher taxonomic diversity. Vegetation type showed no significant influence on either functional or taxonomic diversity (*P* > .05; Fig. 1b and d; Fig. S2a–c). PCA of KEGG ortholog abundances revealed clear depth-based separation of functional gene composition (Fig. 2). PC1 and PC2 explained 51.8% and 18.6% of variance, respectively. Surface and subsurface samples formed distinct clusters (R^2^ = 0.182, *P* < .001), while vegetation had no significant effect (*P* = .923). Surface samples showed broader dispersion across both axes, indicating higher functional heterogeneity, whereas subsurface communities were more compositionally constrained. Squared PCA loadings identified organic N transformation (38%) and N fixation (21%) as dominant contributors to PC1, defining the primary axis of depth-related variation. N fixation (34%) and denitrification (30%) primarily structured PC2, while DNRA and nitrification each accounted for <15% of variance across both components.

### Functional zonation of nitrogen-cycling pathways across peat depths

**Figure 3:**
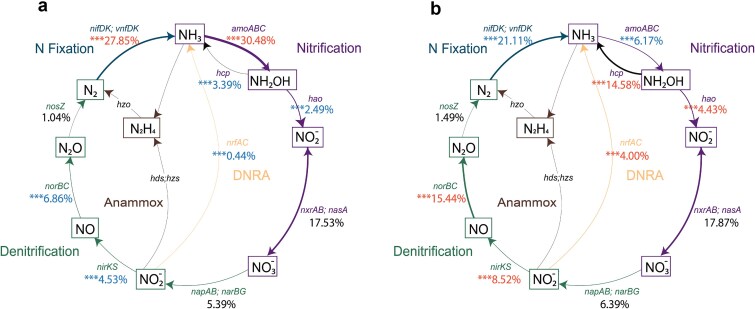
N-cycling gene composition and abundance patterns across peat depth layers. Schematic representation of N-cycling pathways showing the relative abundance of associated genes in (a) surface and (b) subsurface peat layers. Values are expressed as percentages of total N-cycling gene pool analysed in this study, excluding genes associated with organic N hydrolysis and transformation. Only core genes encoding the key enzymes catalyzing major N-cycling pathways were included; percentages therefore represent the relative contribution of each pathway to the overall N-cycling gene pool within each layer. Arrow thickness is proportional to gene abundance within each layer. Percentage values indicate relative abundance, with color coding representing significant differences between depths. Across both panels, red text denotes significantly higher abundance compared with the other depth layer, blue text denotes significantly lower abundance, and black text indicates no significant difference (^***^P < .001). Pathways are distinguished by color. Purple (nitrification), green (denitrification), orange (DNRA), brown (ANAMMOX), and blue (N fixation). Key genes for each transformation are labeled in italics. Brown arrows indicate ANAMMOX pathways (*hds, hzs, hzo*) that were below detection limits in this study.

**Figure 4:**
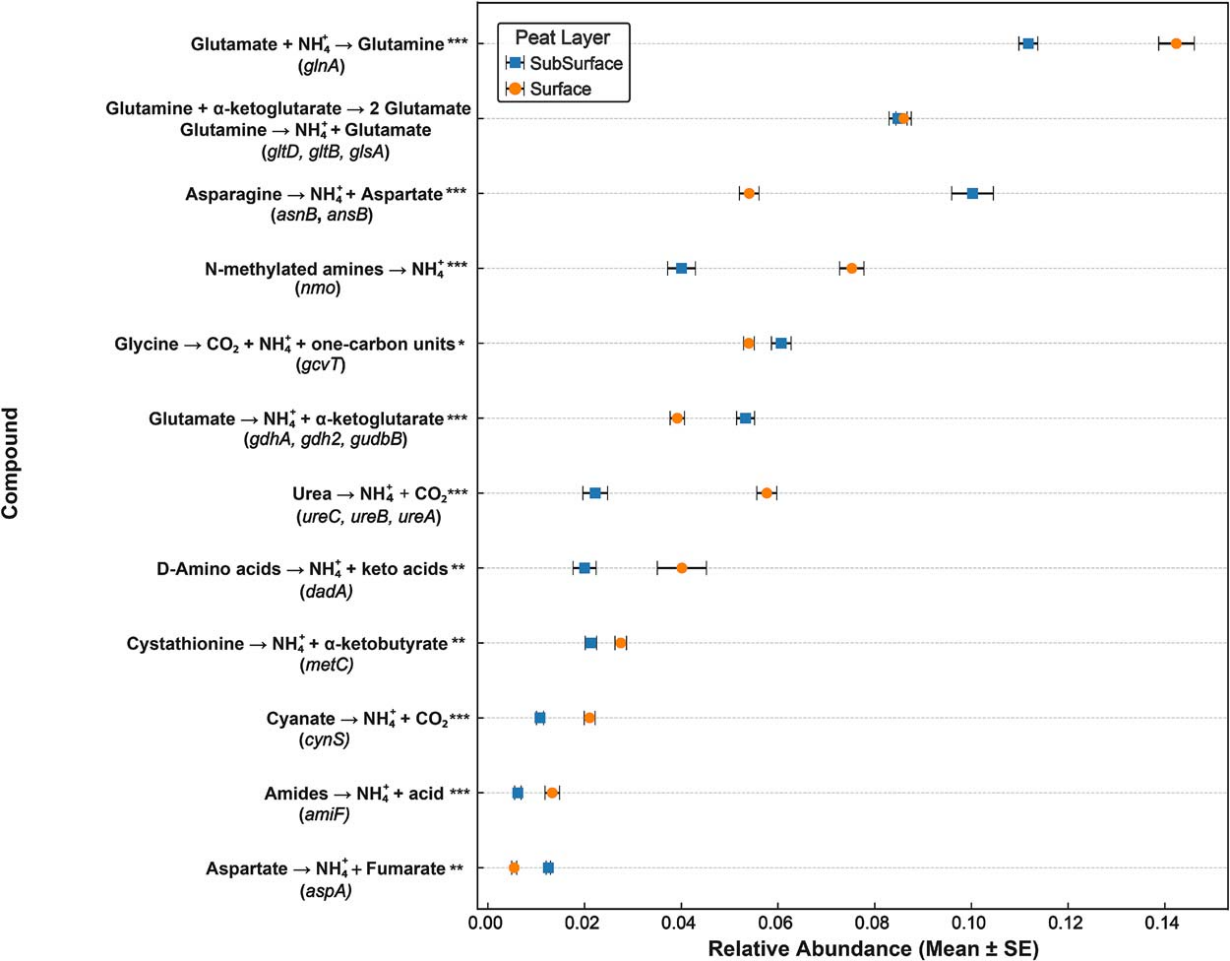
Abundance of genes involved in organic N hydrolysis and transformation pathways across peat depth layers. Gene abundances are expressed relative to the total number of KEGG-annotated genes in each sample, allowing comparison of their contribution of overall microbial metabolic potential. Data points represent mean relative abundance (± standard error) for surface (orange) and subsurface (blue) samples. Each pathway shows the specific enzymatic reaction and associated gene(s) in parentheses. Statistical significance between depth layers is indicated by asterisks (^*^P < .05, ^**^P < .01, ^***^P < .001). Pathways are ordered by decreasing relative abundance.

**Figure 5:**
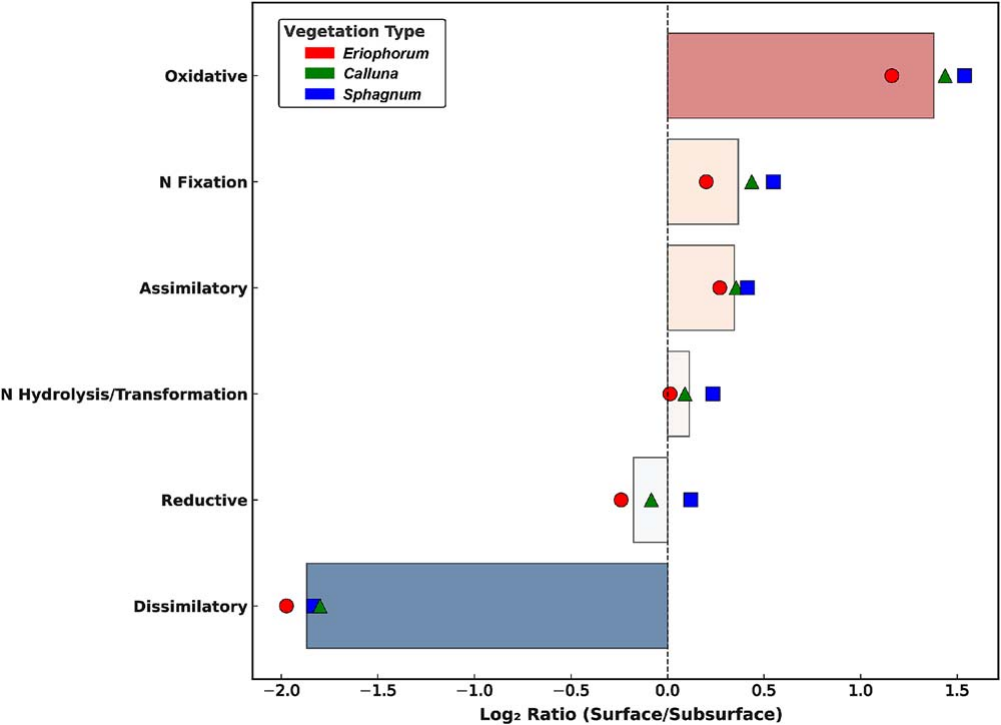
Log_2_-transformed surface-to-subsurface abundance ratios of N-cycling functional pathways across vegetation types. The analysis includes all 70 detected N-cycling genes encompassing N fixation, nitrification, denitrification, DNRA, ANAMMOX, and organic N transformation. Horizontal bars show mean log_2_(surface/subsurface) ratios for each N-cycling pathway, where positive values indicate surface enrichment and negative values indicate subsurface enrichment. The dashed vertical line at zero represents equal abundance between depths. Individual data points represent samples from different vegetation types, *Eriophorum* (red circles), *Calluna* (green triangles), and *Sphagnum* (blue squares). Background shading intensity corresponds to the magnitude of depth stratification, with darker shading indicating stronger layer-specific enrichment. Pathways are ordered from strongest surface enrichment (top) to strongest subsurface enrichment (bottom).

Functional zonation was evident across depth gradients, with clear separation between aerobic surface processes and anaerobic subsurface metabolism (Fig. 3). The vertical stratification of N-cycling processes indicates clear metabolic partitioning across peat depth. Surface peat was characterized by enrichment of oxidative pathways such as nitrification and N fixation, whereas subsurface peat showed greater representation of reductive processes including denitrification and DNRA. Supporting this pattern, genes associated with intermediate denitrification steps displayed clear depth-related differences. Genes encoding nitrite reduction and NO reduction were significantly more abundant in subsurface communities, while genes responsible for N_2_O reduction did not differ significantly across depths or vegetation types (Fig. S3). Organic N transformation pathways also showed depth-specific differentiation despite overall stability across environmental gradients (Fig. 4). Surface-enriched genes included those for N-methylated amine (*nmo*), urea (*ureC/B/A*), D-amino acid (*dadA*), cystathionine (*metC*), cyanate (*cynS*), and amide (*amiF*) degradation, reflecting utilization of labile, N-rich substrates. Subsurface-enriched genes targeted asparagine (*asnB, ansB*), glycine (*gcvT*), and aspartate (*aspA*) metabolism, indicating adaptation to more stable, reprocessed organic compounds. Glutamate/glutamine cycling also diverged by depth, which indicates surface peat favored NH_4_^+^ assimilation via glutamine synthetase (*glnA*), while subsurface layers showed enrichment of glutamate dehydrogenases (*gdhA, gdh2, gudB*) involved in N release and internal recycling. Log_2_-transformed surface-to-subsurface ratios quantified depth enrichment patterns (Fig. 5). Oxidative pathways showed strongest surface enrichment (log_2_ ≈ +1.5), while DNRA showed strongest subsurface enrichment (log_2_ ≈ −1.9). N fixation and assimilatory processes were surface-enriched (log_2_ ≈ +0.3 to +0.5), reductive processes were moderately subsurface-enriched (log_2_ ≈ −0.2), and organic N transformation showed minimal depth preference (log_2_ ≈ 0). Vegetation type did not significantly alter these stratification patterns, though *Sphagnum*-dominated plots showed a consistent tendency toward slightly stronger surface enrichment of oxidative processes, while *Eriophorum* plots showed more even distribution between depths (Table S8).

### Microbial taxa underpinning nitrogen-cycling in peat profiles

**Figure 6:**
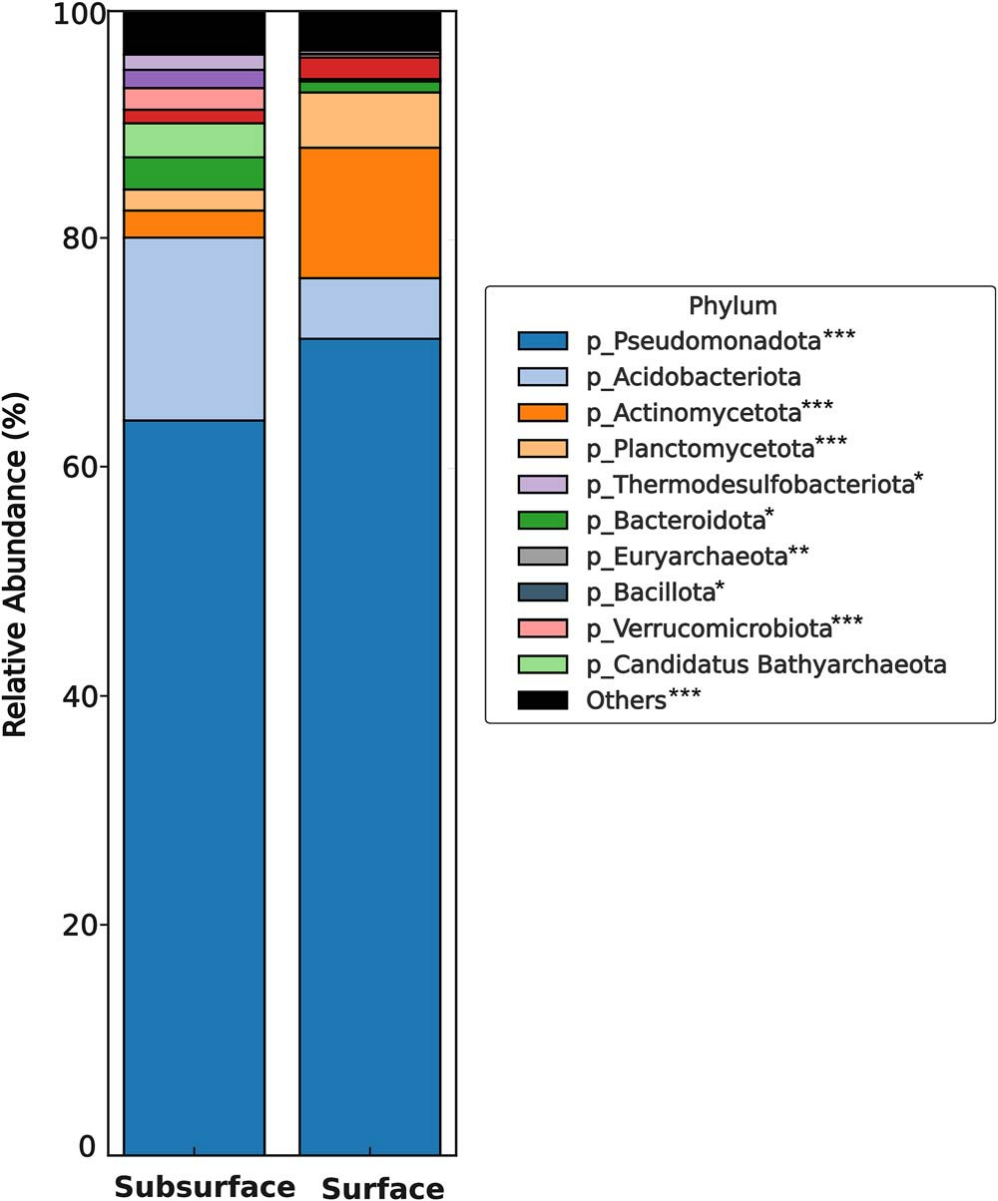
Taxonomic composition of microbial communities associated with N-cycling pathways across peat depth layers. Taxonomic assignments are based on read-level annotations using the NCBI-nr database. Stacked bar charts show the relative abundance of the top 10 phyla involved in N-cycling. Asterisks indicate significant differences between depths (^*^P < .05, ^**^P < .01, ^***^P < .001).

Taxonomic composition shifted markedly with depth (Fig. 6). Pseudomonadota dominated both layers (71.3% surface, 64.2% subsurface; *P* < .001), with significant surface enrichment of Actinomycetota (11.4% vs. 2.4%), Planctomycetota (4.8% vs. 1.8%), and Euryarchaeota (1.93% vs. 1.18%) (all *P* < .01). Subsurface samples were enriched in Thermodesulfobacterota (2.81% vs. 0.95%), Bacteroidota (2.98% vs. 0.22%), and Verrucomicrobiota (1.59% vs. 0.07%) (all *P* < .05). The relative abundance of dominant phyla for all individual samples is provided in Table S9.

**Figure 7:**
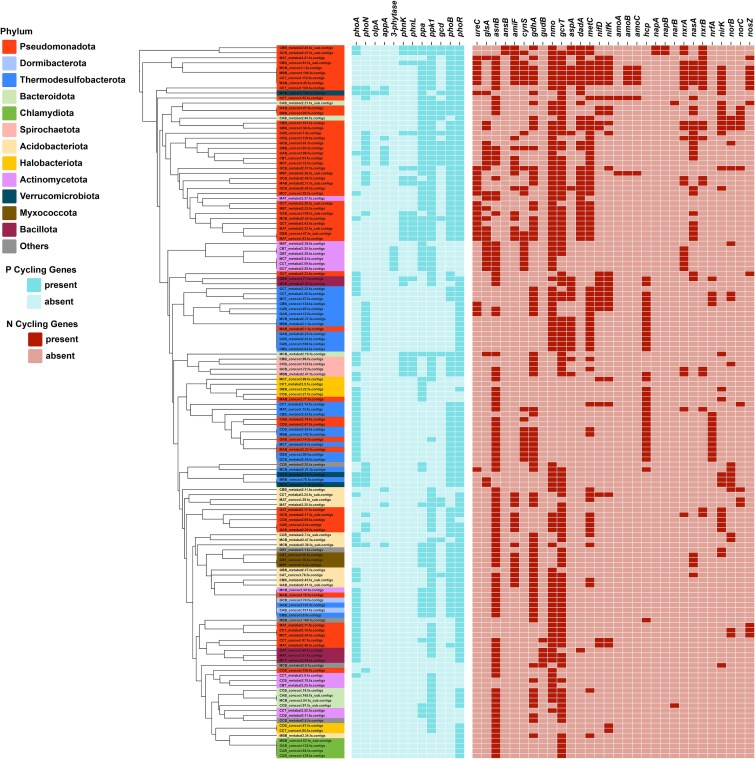
Phylogenetic distribution and N-cycling gene content of MAGs from peat samples. A total of 140 high-quality MAGs are displayed in a phylogenetic tree with taxonomic affiliation indicated by phylum-level color coding. The heatmap shows the presence or absence of key phosphorus-cycling genes (left panel, blue) and nitrogen-cycling genes (right panel, red) within each MAG. Cyan and red cells indicate gene presence, whereas white cells indicate absence. MAGs are ordered based on hierarchical clustering of abundance profiles using Bray–Curtis dissimilarity. Gene names are grouped by functional pathway and labeled accordingly. The key P- and N- cycling genes included in the analysis are summarized in Tables S7 and S10, respectively.

MAG reconstruction yielded 140 high-quality genomes (Fig. 7). Consistent with the taxonomic composition inferred from metagenomic profiling, the majority of MAGs belonged to the dominant bacterial phyla Pseudomonadota (*n* = 48), Thermodesulfobacterota (*n* = 29), Actinomycetota (*n* = 14) and Acidobacteriota (*n* = 13). Functional annotation revealed that many genomes encoded genes associated with both N and P cycling, indicating widespread metabolic potential for coupled nutrient transformations across diverse microbial lineages. N transformation genes were broadly distributed among bacterial MAGs. Genes involved in organic N metabolism, including *asnB, gcvT, aspA*, and *gdhA*, were detected in a large proportion of genomes, indicating widespread genomic potential for amino-acid turnover and organic N transformation in peat microbial communities. Genes associated with N fixation (*nifD* and *nifK*) occurred in a subset of MAGs, primarily affiliated with Pseudomonadota and Thermodesulfobacterota, while ammonia oxidation genes (*amoA, amoB, amoC*) were restricted to Pseudomonadota MAGs, consistent with the established role of this lineage in nitrification. Genes involved in nitrate and nitrite transformations, including *nasA, napA, napB, narB, nxrA, nxrB, nrfA, nirK, norB, norC*, and *nosZ*, were distributed across diverse bacterial phyla. The key N-cycling genes included in the MAG analysis are summarized in Table S10. P cycling genes were also widespread across the reconstructed genomes. Nearly all MAGs encoded genes involved in P acquisition and regulation, particularly *phoB* and *phoR*, suggesting that regulatory responses to phosphate limitation are common across peatland microorganisms. Genes involved in polyphosphate metabolism and intracellular P storage, including *ppk1* and *ppa*, were also frequently detected, while genes associated with organic phosphorus mineralization (OPM) and phosphonate utilization (e.g. *phoA, phoN, phnK*, and *phnL*) occurred in a subset of genomes across multiple bacterial lineages.

### Environmental controls of nitrogen-cycling genes

**Figure 8:**
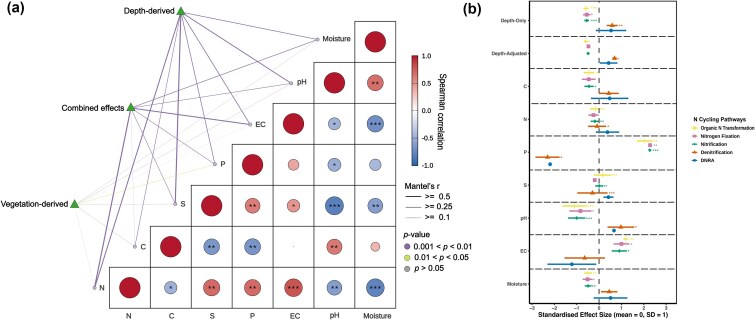
Environmental drivers of N-cycling pathway abundance in peat soils. (a) Correlation matrix showing Spearman correlation coefficients among environmental variables, with circle size proportional to correlation strength and color indicating direction (red = positive, blue = negative). Statistical significance is indicated by asterisks displayed on the circles (^*^P < .05, ^**^P < .01, ^***^P < .001). Mantel test results showing the relative effects of depth, vegetation, and their combined influence on physicochemical properties. Line thickness represents correlation strength (Mantel’s r) and color indicates statistical significance. (b) Standardized effect sizes of environmental predictors on N cycling pathway abundances derived from linear mixed-effects models. Each predictor was evaluated in a separate model with core identity included as a random effect to account for paired samples from the same peat core. Points represent regression coefficients standardized by z-scaling (mean = 0, SD = 1) within each pathway to facilitate comparison of predictor strength, and horizontal bars indicate 95% confidence intervals. Statistical inference is based on the original model coefficients, whereas the standardized values are shown for visual comparison of effect sizes across pathways.

Depth was the primary determinant of peat physicochemistry (Fig. 8a). Mantel tests showed significant depth effects on all measured variables, with strongest correlations for total S (r = 0.713, *P* < .01), total N (r = 0.602, *P* < .01), and pH (r = 0.526, *P* < .01). Moderate associations were found for electrical conductivity, total P, total C, and moisture (r = 0.280–0.427, *P* < .01). Vegetation alone showed no significant correlations with any variable (all *P* > .05) except P (*P* < .05). Combined depth-vegetation effects enhanced associations for total N (r = 0.706, *P* < .001) and electrical conductivity (r = 0.612, *P* < .01) but weakened correlations for S, P, pH, and moisture relative to depth alone. Spearman correlations revealed co-variation among nutrient elements. N was positively correlated with P and S (r = 0.69–0.70, *P* < .01), indicating coupled dynamics in nutrient accumulation. Total C showed negative correlations with nutrient pools (r = −0.49 to −0.68, *P* < .05). Electrical conductivity correlated positively with N (r = 0.79, *P* < .001) and negatively with moisture (r = −0.71, *P* < .001). Peat pH was positively correlated with C (r = 0.63–0.67, *P* < .01) and negatively correlated with N, P, and S availability (r = −0.61 to −0.81, *P* < .05).

Linear mixed-effects models were used to evaluate relationships between environmental variables and N cycling pathways, with core identity included as a random effect. Coefficient estimates and significance were interpreted from the original model outputs, while standardized effect sizes are presented in Fig. 8b to facilitate comparison of predictor strength across pathways. Depth had contrasting effects across N cycling pathways. In the depth-only model, denitrification increased significantly with depth (β = 7.26, *P* < .05), whereas nitrification (β = −4.66, *P* < .001), N fixation (β = −4.96, *P* < .05), and organic N transformation (β = −5.33, *P* < .001) all declined with depth. When environmental variables were incorporated in the depth-adjusted model, the positive association between depth and denitrification became stronger (β = 11.53, *P* < .05), while the negative association with organic N transformation remained significant (β = −5.81, *P* < .05). In contrast, the previously significant depth effects observed for nitrification and N fixation became non-significant after accounting for environmental variables, suggesting that part of the apparent depth-related variation in these pathways reflects underlying environmental gradients within the peat profile rather than depth alone.

Environmental drivers varied across N cycling pathways. Nitrification showed strong positive associations with P (β = 95.40, *P* < .01), S (β = 15.12, *P* < .01), and EC (β = 47.07, *P* < .05), while the negative relationships were observed with pH (β = −20.53, *P* < .001), moisture (β = −2.04, *P* < .01), N availability (β = −8.35, *P* < .05), and C (β = −0.90, *P* < .05). In contrast, denitrification was negatively associated with N (β = −12.03, *P* < .05), P (β = −109.63, *P* < .05), and S (β = −28.45, *P* < .001), whereas pH showed a positive relationship with this pathway (β = 22.98, *P* < .05). Distinct patterns were also observed for other N cycling pathways. N fixation was positively associated with P (β = 128.33, *P* < .01) and EC (β = 68.06, *P* < .05), but declined with increasing pH (β = −18.39, *P* < .05) and moisture (β = −3.28, *P* < .05). Organic N transformation showed positive relationships with P (β = 80.53, *P* < .01), N (β = 8.15, *P* < .01), S (β = 18.70, *P* < .001), and EC (β = 52.62, *P* < .01). While C (β = −1.10, *P* < .01), pH (β = −21.83, *P* < .001), and moisture (β = −2.52, *P* < .01) were negatively associated with pathway abundance. In comparison, DNRA showed comparatively weaker relationships with most environmental variables. Notably, P effect sizes were the largest among environmental predictors. The magnitude of its regression coefficients, particularly for nitrification, N fixation, and organic N transformation, were substantially greater than those observed for other variables, indicating that depth-related variation was partially masking P’s regulatory importance when assessed independently.

**Figure 9:**
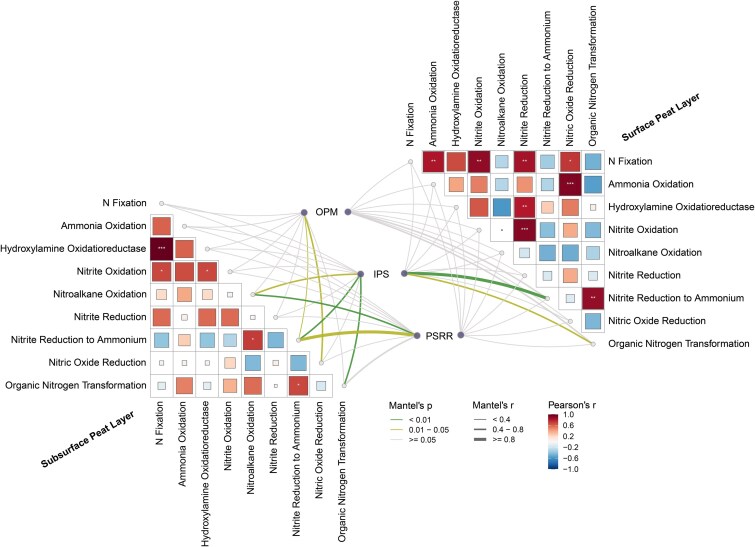
Correlations between P-cycling strategies and N-cycling pathways in subsurface peat (left) and surface peat (right). The heatmaps show pairwise spearman correlations among N-cycling pathways, including N fixation, ammonia oxidation, hydroxylamine oxidoreductase, nitrite oxidation, nitroalkane oxidation, nitrite reduction, nitrite reduction to ammonium, NO reduction, and organic nitrogen transformation. Color intensity indicates the direction and strength of the correlation coefficient, with red indicating positive correlations and blue indicating negative correlations. Asterisks denote significant correlations among N-cycling pathways. The central network shows Mantel test correlations between P-cycling gene categories and N-cycling pathways. P-cycling genes were grouped into OPM, IPS, and PSRR; the genes included in each category are listed in Table S7. Line color indicates Mantel test significance (green, *P* < .01; yellow, .01–.05; gray, ≥.05), and line thickness indicates Mantel correlation strength (r). Together, the figure compares the internal correlation structure of N-cycling pathways and their associations with P-cycling strategies between subsurface and surface peat communities.

Mantel network analysis revealed clear differences in the associations between P cycling strategies and N cycling pathways across peat layers (Fig. 9). In the surface peat, only inorganic phosphorus solubilization (IPS) showed significant correlations with nitroalkane oxidation (r = 0.85, *P* < .01) and organic N transformation (r = 0.72, *P* < .05). In contrast, the subsurface peat exhibited a greater number and diversity of significant correlations between P acquisition strategies and N cycling processes. Organic P mineralization was significantly associated with nitrite reduction to ammonium (r = 0.33, *P* < .05) and NO reduction (r = 0.50, *P* < .05). IPS showed significant correlations with nitroalkane oxidation (r = 0.51, *P* < .05), nitrite reduction to ammonium (r = 0.59, *P* < .01), and organic N transformation (r = 0.62, *P* < .01). In addition, PSRR showed strong associations with pathways of nitroalkane oxidation (r = 0.68, *P* < .001) and nitrite reduction to ammonium (r = 0.81, *P* < .05).

### Community assembly and co-occurrence networks

Community assembly mechanisms differed markedly between depths. NST analysis indicated deterministic assembly in surface samples (NST = 44.4%), while subsurface samples showed predominantly stochastic assembly (NST = 58.3%) (Fig. S4). Co-occurrence network analysis revealed depth-dependent coordination patterns (Figs S5 and S6). Surface functional gene networks were more complex and cohesive (70 nodes, 313 edges, network density = 0.13, average degree = 8.94) compared to subsurface networks (48 nodes, 40 edges, network density = 0.035, average degree = 1.67), indicating tighter metabolic coordination in the surface peat layer. Conversely, MAG-based taxonomic networks showed the opposite pattern, where subsurface communities formed denser networks (137 nodes, 2205 edges, network density = 0.237, average degree = 32.19) than surface communities (136 nodes, 1761 edges, network density = 0.192, average degree = 25.9), suggesting that taxonomic cooperation through metabolite exchange and syntrophy compensates for reduced functional versatility in oligotrophic, anoxic subsurface conditions. These contrasting network topologies support the functional-taxonomic decoupling observed in diversity analyses.

## Discussion

### Depth-driven stratification of nitrogen-cycling mechanisms in bog ecosystems

Unsurprisingly, depth is the primary driver of N-cycling stratification in this blanket bog, governing both input processes (N fixation, organic N transformation) and downstream pathways (nitrification, DNRA, denitrification) [23]. The pronounced functional zonation reflects adaptation to contrasting redox and substrate availability gradients across the peat profile. Surface peat showed elevated N fixation potential (28% of N cycling gene pool vs. 21% subsurface), consistent with greater diazotroph capacity to exploit anoxic microsites (water-filled pores, aggregate interiors) where atmospheric N_2_ and nutrients are both accessible. The vertical differentiation of organic N transformation genes highlights distinct microbial strategies for organic N utilization. Surface communities were enriched in genes targeting labile, N-rich substrates (urea, N-methylated amines, D-amino acids) typically derived from fresh plant inputs, microbial turnover, and rapid mineralization processes characteristic of dynamic, oxygenated surface conditions [55–57]. Conversely, subsurface enrichment of genes for asparagine, glycine, and aspartate metabolism indicates adaptation to more stable compounds originating from reprocessed organic matter, reflecting selective N use under anaerobic, resource-limited conditions [58, 59]. The glutamate-glutamine pathway exhibited clear depth-dependent divergence. Surface enrichment of *glnA* (glutamine synthetase) suggests microbial preference for ammonium assimilation and anabolic N retention [55], while subsurface enrichment of *gdhA, gdh2*, and *gudB* (glutamate dehydrogenase) reflects a catabolic strategy focused on N release and internal recycling. Together, these patterns indicate that surface communities exploit reactive N pools through assimilatory pathways, whereas subsurface microbes rely on conservative, recycling-oriented processes. This functional differentiation has important implications for N retention versus loss from different peat layers. Depth also governed downstream inorganic N-cycling pathways. Surface layers were dominated by oxidative processes, particularly ammonia oxidation (30% of gene pool), highlighting high nitrification potential in oxygen-rich microenvironments. In contrast, deeper layers were enriched in reductive and dissimilatory pathways, with denitrification genes comprising 32% of the subsurface gene pool and DNRA showing 10-fold enrichment relative to surface layers. Community-level gene abundance patterns further revealed potential bottlenecks in terminal denitrification steps. The near-twofold greater abundance of genes associated with NO and N_2_O production in subsurface communities (Figs 3 and S3), combined with limited N_2_O reduction capacity, points to an imbalance in the final steps of the denitrification pathway. This pattern indicates that the genetic potential for N_2_O consumption does not increase in parallel with N_2_O-producing steps, implying that denitrification pathways may be incomplete in subsurface peat communities and potentially favoring N_2_O accumulation under waterlogged conditions. While this imbalance can be linked with a greater potential for N_2_O production in subsurface peat [24, 60], such relationships should be interpreted with caution because microbial genetic potential does not necessarily translate directly into ecosystem-scale N_2_O emissions, which are often strongly regulated by environmental conditions [61].

The functional-taxonomic decoupling observed across depth has important mechanistic implications. Surface communities, experiencing dynamic environmental conditions (fluctuating moisture, temperature, oxygen availability) and continuous resource inputs, assemble deterministically around coordinated metabolic strategies. This is reflected in tight functional gene co-occurrence networks (average degree = 8.94) despite moderate taxonomic diversity (136 MAGs). Conversely, stable subsurface conditions favor stochastic assembly (NST = 58.3%) and taxonomic cooperation over functional specialization. Dense taxonomic networks in the subsurface (2205 edges vs. 1761 in surface) likely reflect metabolite exchange, syntrophy, and niche complementarity that compensate for reduced functional versatility (average degree = 1.67 for functional networks) in oligotrophic, energy-limited conditions [62, 63]. This decoupling pattern has important implications for predicting ecosystem responses to environmental change, where functional redundancy in surface layers may confer resilience to disturbance, while taxonomic cooperation in subsurface layers suggests vulnerability to disruptions in syntrophic partnerships.

### Environmental regulation of nitrogen-cycling mechanisms

Although peat depth strongly structures soil physicochemical conditions, its direct influence on microbial N-cycling pathways was partly mediated by underlying environmental gradients. The disappearance of depth effects for nitrification and N fixation after environmental variables were included in the models indicates that vertical variation in these pathways largely reflects depth-related changes in soil chemistry rather than spatial position itself. In contrast, denitrification remained positively associated with depth even after accounting for environmental variables, suggesting that additional depth-dependent factors, such as redox dynamics or oxygen availability, may influence this pathway. The pathways examined here showed distinct environmental sensitivities. Nitrification and organic N transformation were strongly associated with multiple chemical variables, including P, S, and EC, while declining under higher pH, moisture, N, and C availability. N fixation also showed strong positive responses to P availability, reinforcing the importance of P supply for processes regulating N inputs to peat ecosystems. In contrast, denitrification responded differently, showing negative associations with several nutrients but a positive relationship with pH, suggesting that redox conditions and electron-acceptor availability may play an important role in regulating this pathway. Across these pathways, P showed the largest regression coefficients among environmental predictors, indicating that P supply appears to exert a strong constraint on microbial functional potential. By comparison, DNRA showed comparatively weak relationships with the measured environmental variables, indicating that this pathway may be governed by factors not captured by bulk soil chemistry. DNRA activity often occurs under highly localized anaerobic conditions, and its variability may therefore depend more strongly on microscale redox heterogeneity, organic substrate quality, or microbial interactions within peat microenvironments.

Our results highlight that P availability is a key predictor of microbial N-cycling gene abundance in this ombrotrophic blanket bog, challenging the traditional paradigm that N limitation dominates peatland biogeochemistry and thereby supporting growing evidence that P availability can regulate microbial N cycling even in ecosystems where total N pools are large [8–10]. Increased P availability has been shown to stimulate microbial N-cycling activity, as supported by previous experimental studies [64, 65]. This interpretation is consistent with work showing that P availability accelerates N cycling in acidic soils, and also acts as a significant predictor of denitrification gene abundance [66]. P availability is known to exert strong control over microbial N cycling through stoichiometric and enzymatic constraints [67].

From a genomic perspective, our results further support this coupling between P acquisition and N transformation processes. In the surface peat layer, where P availability is relatively higher, only microbial inorganic P solubilization pathways showed correlations with N cycling mechanisms. In contrast, in the subsurface peat, genes associated with the PSRR had broad connectivity with NH_4_^+^ and NO_3_^−^ production pathways, reflecting coordinated regulatory responses to P limitation. In addition, associations between organic P mineralization and inorganic P solubilization with N cycling pathways were substantially stronger in the subsurface peat. This pattern suggests that coupling between P acquisition and N transformations becomes more pronounced under deeper peat conditions where nutrient availability and redox environments differ. Together with genome-resolved evidence showing frequent co-occurrence of P acquisition genes and N metabolism genes within individual MAGs, these results highlight a potential ecological linkage between microbial P and N cycling.

This coupling can be explained by the metabolic constraints imposed under P-limited conditions. When P bioavailability is low, microorganisms must invest additional metabolic resources in P acquisition to sustain growth, particularly through increased production of extracellular phosphatases that hydrolyze organic P compounds into bioavailable phosphate [68]. Because phosphatases are N-rich proteins, their synthesis requires substantial N investment, creating a direct biochemical linkage between P acquisition and microbial N metabolism [69]. Consequently, P limitation can influence N cycling by redirecting microbial N resources toward enzyme production and nutrient acquisition processes. A complementary framework for interpreting these depth-dependent patterns is the trade-off between microbial C use efficiency and nutrient-acquiring extracellular enzyme activity under reduced oxygen conditions. Under oxygen-limited conditions, microorganisms may allocate a greater proportion of resources toward maintenance and nutrient acquisition rather than growth, leading to increased biomass-specific enzyme production and reduced growth efficiency [70]. In peatland systems, oxygen limitation has been shown to suppress oxidative enzyme activity while altering hydrolytic and phosphatase-mediated processes, thereby influencing nutrient acquisition strategies [71, 72]. Within this framework, the greater number and strength of associations between P acquisition strategies and N-cycling pathways observed in subsurface peat may reflect increased microbial investment in nutrient acquisition under energy-limited, anoxic conditions rather than enhanced growth efficiency. However, because extracellular enzyme activities and microbial growth efficiency were not directly measured in this study, these interpretations should be regarded as plausible mechanistic explanations.

Total N availability acted as a suppressing factor for both nitrification and denitrification (negative β coefficients). This pattern is consistent with N saturation theory, where excess N accumulation can inhibit specific N-cycling processes when other nutrients become limiting [73]. Similar nutrient-enrichment effects on N-cycling microbial communities have been documented in forest and grassland ecosystems [74, 75]. In peatland systems, this effect may be amplified because N pools are predominantly organic rather than bioavailable inorganic forms. When organic N accumulates faster than microbial processing capacity, it may suppress classical nitrification–denitrification pathways while promoting direct organic N recycling. Collectively, these findings provide strong genetic evidence for the regulatory role of N:P stoichiometry in shaping microbial function and biogeochemical cycling [9, 65, 76], with lower N:P ratios associated with more active N transformations and potentially greater N loss from the ecosystem.

The minimal vegetation effect on microbial N-cycling structure warrants careful interpretation and likely reflects the ecological reality of blanket bog vegetation organization. While plots were classified by dominant species, all contained mixed assemblages of *Sphagnum, Eriophorum, Calluna*, and other species. Root systems intertwine extensively in peat profiles [30], creating spatially heterogeneous rhizosphere effects, and vegetation composition shifts over annual to decadal timescales [77], further homogenizing microbial communities at the spatial scale sampled. Previous studies reporting strong vegetation effects on peatland microbial communities often employed experimental designs with true monocultures [28], mesocosm controls [29], or focused on specific plant-microbe associations [76]. In natural, mixed-species blanket bogs, depth-driven physicochemical gradients, particularly redox stratification and nutrient availability, appear to override subtle vegetation influences on bulk microbial function. Vegetation alone did not significantly structure nutrient pools in our system (Mantel test *P* > .05), although depth-vegetation interactions showed minor effects (r = 0.706 for N, r = 0.612 for EC), consistent with recent observations that plant functional type effects are most apparent when considered alongside vertical gradients [78]. Nonetheless, subtle trends were observable. Bryophyte-associated communities showed slight tendency toward greater N fixation and oxidative process potential (Fig. 5, Table S8), consistent with *Sphagnum*’s capacity to concentrate atmospheric N inputs across photosynthetic tissues [79]. Vascular plant-associated metagenomes showed marginally more even distribution of oxidative versus reductive processes, potentially reflecting deeper rooting depths that create mixed redox microenvironments [80]. However, these patterns were not statistically significant and require validation through expanded replication or manipulative experiments to separate vegetation effects from spatial heterogeneity.

### Taxonomic profiling of nitrogen-cycling microbiome in bog ecosystems

Pseudomonadota emerged as the dominant lineage mediating N-cycling across depths (71.3% surface, 64.2% subsurface for inorganic N-cycling), showing exceptional functional breadth. This phylum encoded key genes for N fixation (*nifD, nifK*), ammonia oxidation (*amoA/B/C*; exclusive to Pseudomonadota), and complete denitrification pathways (*nap, nir, nor, nosZ*), positioning them as central hubs for N input, internal turnover, and loss. Their genomic versatility is consistent with observations of Pseudomonadota dominance and metabolic flexibility across diverse terrestrial and aquatic ecosystems [80–83]. As heterotrophic diazotrophs in this organic-rich, N-limited environment, Pseudomonadota likely rely heavily on reduced organic compounds for energy and C [84]. Thermodesulfobacterota also contributed substantially to N-cycling, harboring genes for anaerobic N fixation in addition to denitrification capacity [85]. The strong subsurface enrichment of this phylum (2.81% vs. 0.95% in surface; *P* < .05) and its representation in 29 MAGs, predominantly from subsurface samples, underscores its importance in mediating both N acquisition and loss in anoxic, nutrient-poor zones. This dual role demonstrates the significance of this often-overlooked phylum in blanket bog biogeochemistry. Beyond these dominant groups, Actinomycetota, Planctomycetota, Bacteroidota, and other lower-abundance lineages contributed scattered N-cycling functions, likely supporting auxiliary or complementary roles within the broader N network. Notably, genes for organic N transformation were nearly ubiquitous across all MAGs, highlighting the critical and universal role of microorganisms in N retention and transformation. This broad distribution suggests that organic N cycling capacity is a fundamental trait across diverse taxa in oligotrophic peatlands, essential for internal N recycling when external inputs are limited. The rarity of canonical nitrite reductases (*nirS* and *nirK*), which were restricted to Pseudomonadota and remained uncommon even within this phylum (Fig. 7), indicates a constrained capacity for denitrification at the nitrite reduction step. Combined with the limited abundance of *nosZ* relative to upstream denitrification genes (*norB* and *norC*), this suggests an imbalance in the terminal step of the pathway. Additionally, given the absence of *nirK/S* genes, there is indication that NO is being produced via alternative pathways, possibly nitrification. These patterns point toward a genetic potential for incomplete denitrification, which may favor N_2_O accumulation rather than complete reduction to N_2_, with important implications for greenhouse gas emissions from blanket bogs [24, 86].

### Advancing understanding of peatland microbial nitrogen-cycling

This study demonstrates the power of depth-stratified shotgun metagenomics for revealing functional and taxonomic organization in peatland ecosystems. Our two-layer design, aligned with the redox transition zone governed by water table fluctuations, captured the fundamental redox boundary structuring microbial metabolism. July sampling during peak activity revealed clear depth-driven stratification that establishes baseline patterns for future investigations. Our MAG-based approach provided taxonomic resolution unavailable through amplicon methods, identifying specific lineages mediating N transformations.

Our results also refine the role of depth in structuring N-cycling potential in ombrotrophic peat. Although depth exerted strong control over peat physicochemistry, its direct effects on N-cycling gene abundance were weakened once P, N, pH, and other environmental variables were included in the models. This indicates that depth does not act as an independent spatial driver, but instead operates primarily through measurable gradients in nutrient availability and acidity, particularly P availability and pH. That is, vertical patterns in microbial N-cycling capacity emerge largely because depth structures the physicochemical environment, rather than because depth itself has an intrinsic effect. This has important implications for predicting how external nutrient inputs will modify depth-related patterns in N cycling. If depth effects are mediated mainly through P availability, then changes in atmospheric deposition or local P enrichment are likely to alter N-cycling capacity in a depth-dependent but mechanistically predictable way. For example, alleviating P limitation in surface layers could enhance nitrification and denitrification disproportionately near the peat surface, potentially decoupling traditional relationships between redox status and N dynamics [86, 87]. Recognizing that depth acts through P and other measured variables therefore provides a more mechanistic basis for forecasting how nutrient loading will reshape vertical patterns of N cycling in ombrotrophic blanket bogs.

Despite these advances, an important uncertainty lies in how far these genomic patterns can be translated into ecosystem-scale rates of N turnover. While our depth-resolved metagenomic approach captured in situ variation across the peat profile, it primarily reflects microbial functional potential rather than realized activity. Upscaling microbial processes from controlled or localized observations to ecosystem function remains challenging, as spatiotemporal variability, plant–soil–microbial feedbacks, and complex interactions among environmental drivers can generate discrepancies between observed gene abundance and actual biogeochemical fluxes [88–90]. In peatlands, this challenge is further amplified by hydrologically driven redox heterogeneity, where water-table position and soil moisture regulate oxygen availability, yet may not precisely predict the spatial distribution of oxic–anoxic interfaces within the peat profile [25, 86]. Accordingly, our results should be interpreted as evidence for depth-structured functional potential and putative controls on microbial N cycling, rather than direct estimates of in situ process rates.

## Supplementary Material

supplementary_material_Shuaizhi_RM_ycag143

## Data Availability

The raw sequence data from this study are available under NCBI BioProject accession number: PRJNA1301652 (https://www.ncbi.nlm.nih.gov/bioproject/PRJNA1301652). Associated BioSample accessions are SAMN50450969–SAMN50450986. The corresponding peat physicochemical data are shown in Table S1.
