# Supplementary material for: Phosphorus availability mediates pathway-specific nitrogen cycling in stratified peatland microbiomes
Source: ISME Commun. 2026 May 23;6(1):ycag143. doi: 10.1093/ismeco/ycag143 (PMC13298671; doi:10.1093/ismeco/ycag143)
Supplement: supplementary_material_Shuaizhi_RM_ycag143 [file supplementary_material_shuaizhi_rm_ycag143.pdf]

Supplemental Information for:

Phosphorus Availability Mediates Pathway-Specific Nitrogen Cycling in Stratified Peatland Microbiomes

Authors: Shuaizhi Guo[1], Niall P. McNamara[2], Gary D. Bending[1], Ryan M. Mushinski[1*]

Author Affiliations:

[1] School of Life Sciences, Gibbet Hill Campus, University of Warwick, Coventry, CV4 7AL, UK

[2] Centre for Ecology and Hydrology, Lancaster Environment Centre, Library Avenue, Bailrigg, Lancaster LA1 4AP, UK

***Corresponding author:** Ryan M. Mushinski [Ryan.Mushinski@warwick.ac.uk]

Mailing Address:

School of Life Sciences

Gibbet Hill Campus

University of Warwick

Coventry CV4 7AL

United Kingdom

Table of Contents

Supplementary Figures **Page Number**

Figure S1: Monthly water table depth fluctuations from 2014 to 2024 .... S3

Figure S2: Taxonomic alpha diversity across peat depth and vegetation types S4

Figure S3: Abundances of N_2O production and consumption genes across vegetation types and depth layers . S6

Figure S4: Normalised Stochasticity Ratio of Functional Gene Communities S7

Figure S5: Co-occurrence Network of Nitrogen Cycling Genes S8

Figure S6: Co-occurrence Network of Metagenome-Assembled Genomes S9

Supplementary Tables

Table S1. Physicochemical properties of peat samples. 10

Table S2. Comparison of physicochemical properties between surface and subsurface peat layers 10

Table S3. Functional genes involved in nitrogen cycling processes 11

Table S4. Spearman correlations between KEGG *amo* genes and NCycDB *pmo* genes 12

Table S5. KEGG-NCycDB genes and functional gene ratio correlations (Spearman). 12

Table S6. KEGG-NCycDB genes and functional gene ratio correlations (Pearson). 12

Table S7. Details on the key functional genes related to phosphorus cycling studied in the present work 12

Table S8. Pairwise Welch t-test comparisons of log_2 surface-to-subsurface ratios of nitrogen cycling processes among vegetation types. 13

Table S9. Relative abundance of dominant microbial phyla carrying nitrogen-cycling genes across peat samples under different vegetation types and depths 14

Table S10. Details on the key functional genes related to nitrogen cycling studied in the present work 15

Supplementary Figures

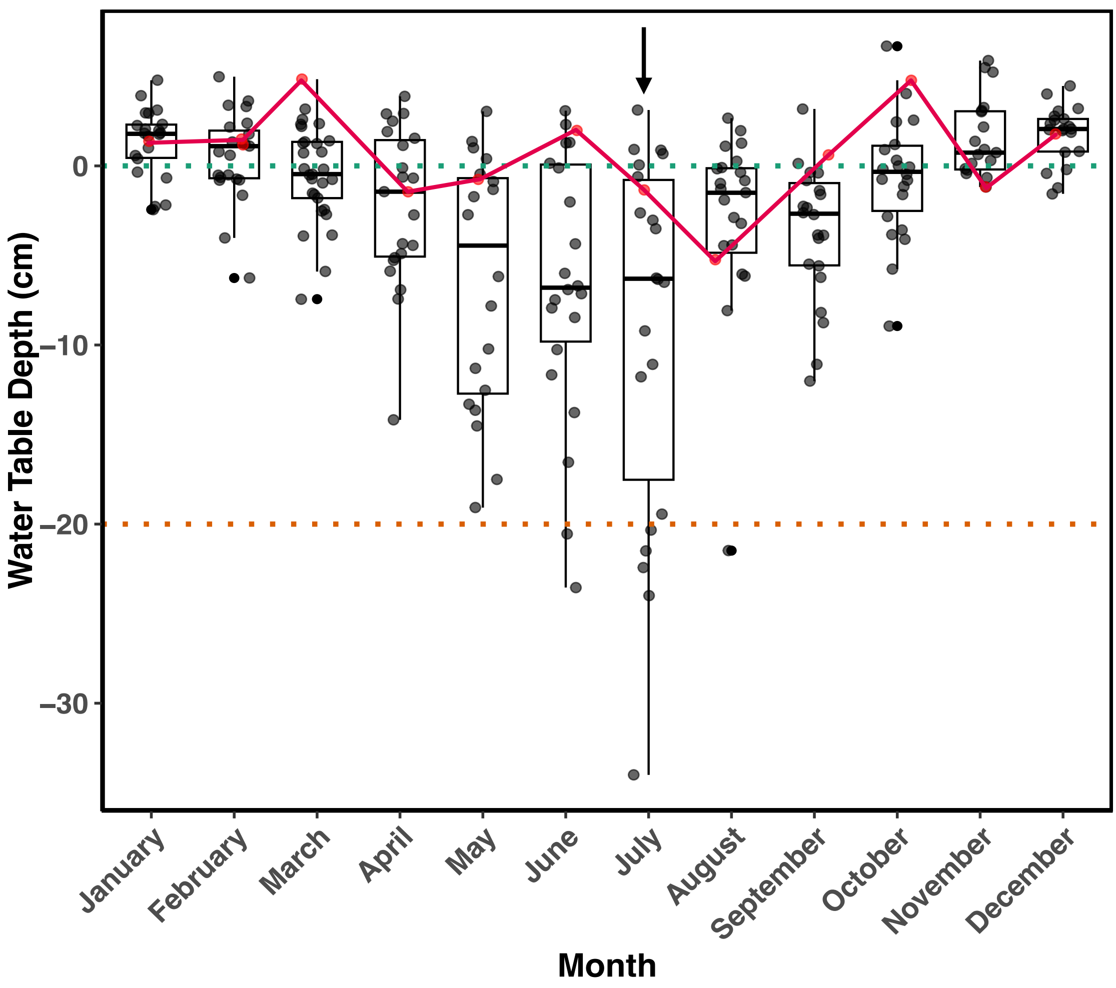

Figure S1. Monthly water table depth fluctuations from 2014 to 2024 at the study site. Red dots and lines represent measurements from 2024, with field sampling conducted in July (indicated by arrow). Negative values indicate water table below the peat surface.

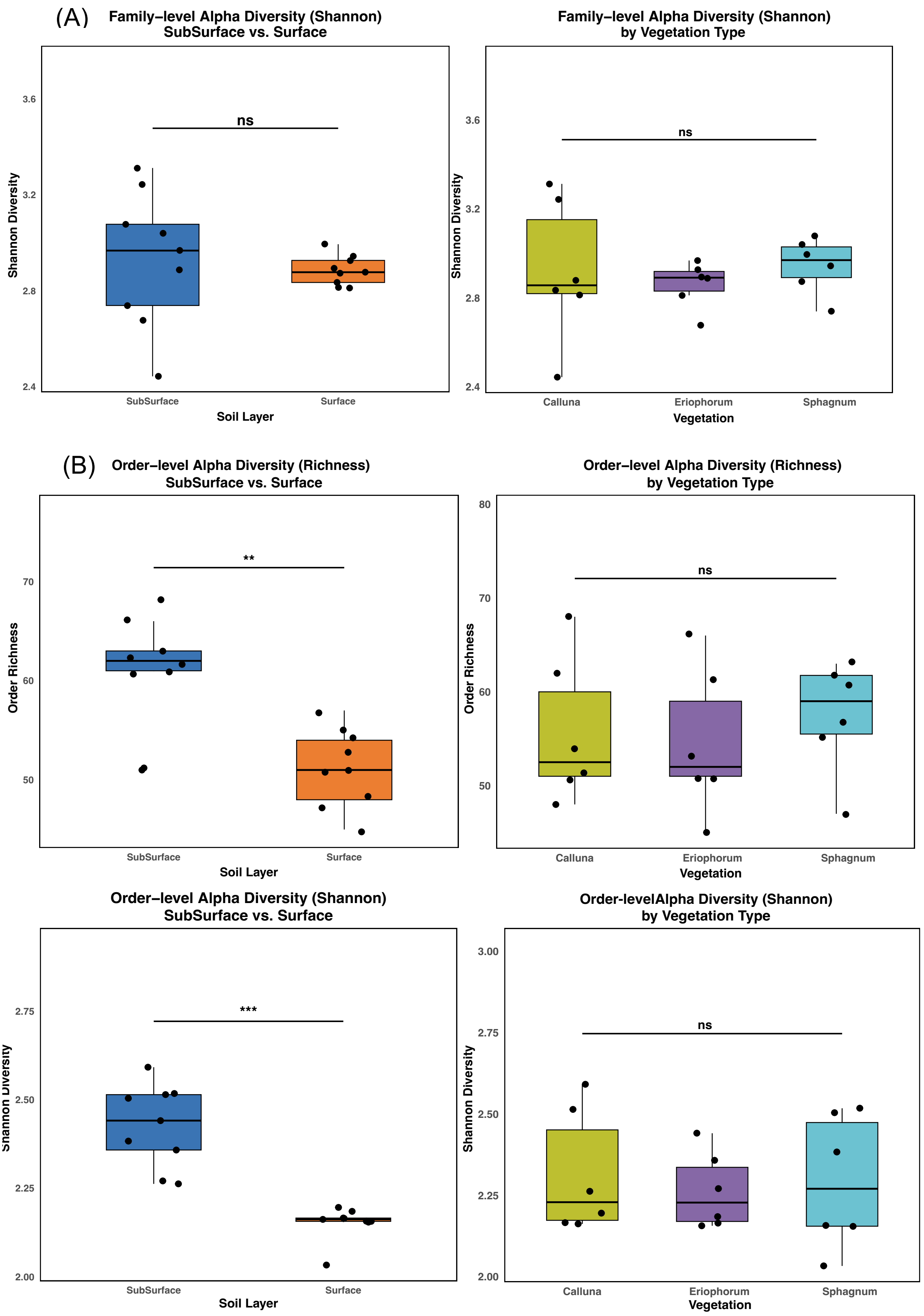
(A)
Family–level Alpha Diversity (Shannon)
SubSurface vs. Surface
ns
Shannon Diversity
3.6
3.2
2.8
2.4
SubSurface
Surface
Soil Layer
Family–level Alpha Diversity (Shannon)
by Vegetation Type
ns
Shannon Diversity
3.6
3.2
2.8
2.4
Calluna
Eriophorum
Sphagnum
Vegetation
(B)
Order–level Alpha Diversity (Richness)
SubSurface vs. Surface
**
Order Richness
70
60
50
SubSurface
Surface
Soil Layer
Order–level Alpha Diversity (Richness)
by Vegetation Type
ns
Order Richness
80
70
60
50
Calluna
Eriophorum
Sphagnum
Vegetation
Order–level Alpha Diversity (Shannon)
SubSurface vs. Surface
***
Shannon Diversity
2.75
2.50
2.25
2.00
SubSurface
Surface
Soil Layer
Order-levelAlpha Diversity (Shannon)
by Vegetation Type
ns
Shannon Diversity
3.00
2.75
2.50
2.25
2.00
Calluna
Eriophorum
Sphagnum
Vegetation

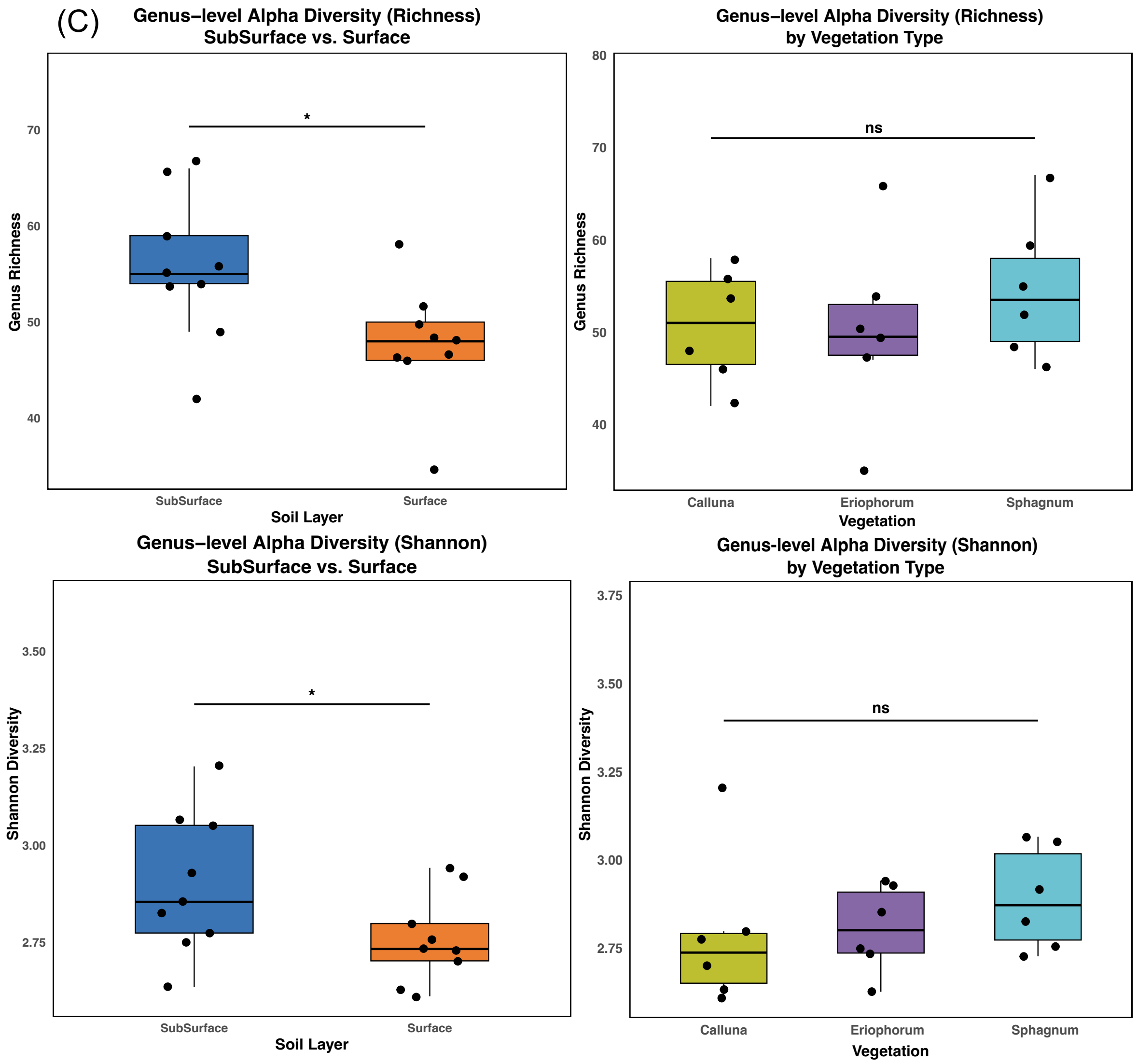

Figure S2. Taxonomic alpha diversity across peat depth and vegetation types at multiple taxonomic resolutions. (A) Family-level Shannon diversity comparing surface and subsurface communities and across vegetation types. (B) Order-level alpha diversity showing richness and Shannon diversity across soil layers and vegetation types. (C) Genus-level alpha diversity showing richness and Shannon diversity across soil layers and vegetation types. Points represent individual samples and boxplots summarise the distribution within each group. Statistical significance is indicated as *p < 0.05 and ***p < 0.001, while “ns” denotes non-significant differences.

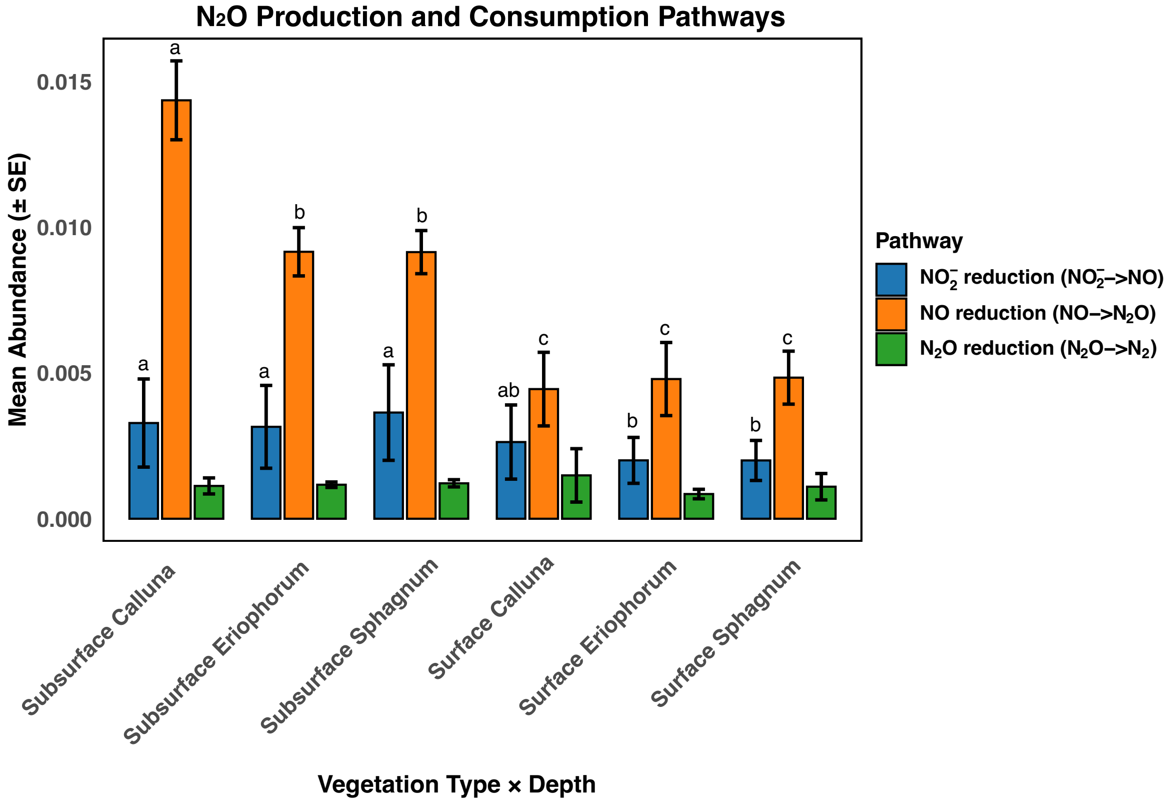

Figure S3. Abundances of genes involved in N_2O production and consumption pathways across vegetation types and depth layers. Bars show mean gene abundance (± SE) for nitrite reduction (*nirS*, *nirK*; NO_2^- → NO), nitric oxide reduction (*norB*; NO → N_2O), and N_2O reduction (*nosZ*; N_2O → N_2) in surface and subsurface peat associated with Calluna, Eriophorum, and Sphagnum. Genes involved in NO_2^- and NO reduction were significantly more abundant in subsurface samples, whereas *nosZ* showed no significant differences across depths or vegetation types. Different letters indicate significant differences among groups ($p < 0.05$).

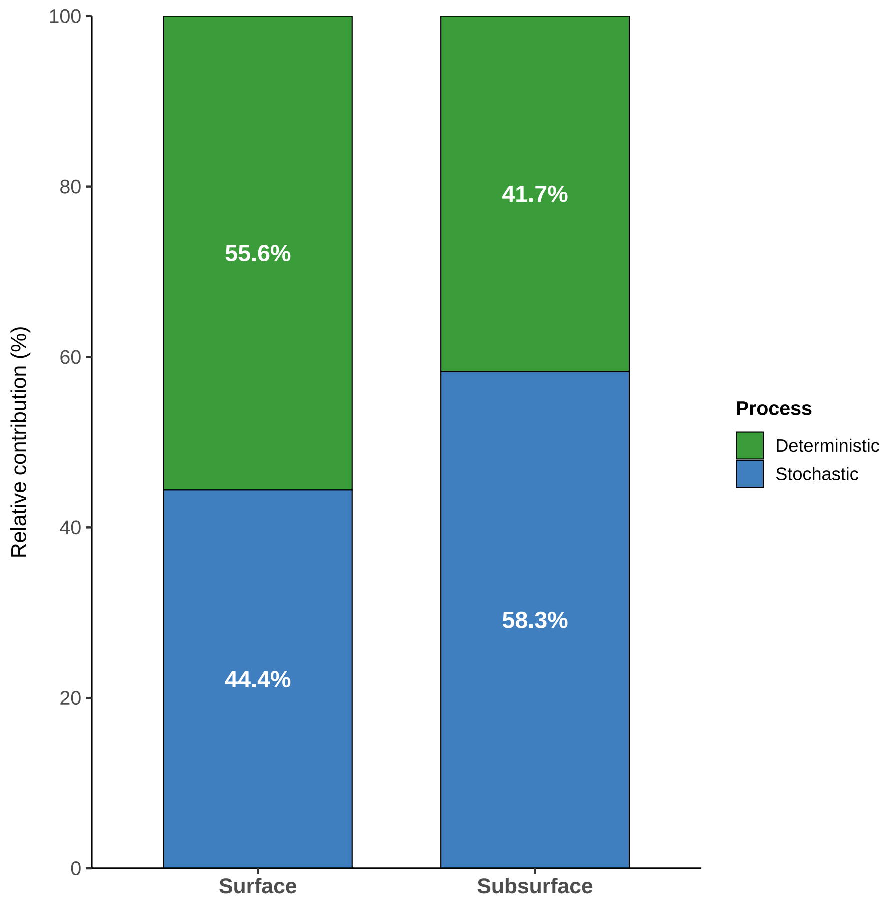

Figure S4. Normalised stochasticity ratio (NST) analysis of nitrogen-cycling gene communities in surface and subsurface peat layers. NST values indicate the relative contribution of stochastic processes to community assembly, with values >50% indicating stochastic dominance and <50% indicating deterministic dominance. Surface communities showed predominantly deterministic assembly (NST = 44.4%), while subsurface communities were governed by stochastic processes (NST = 58.3%).

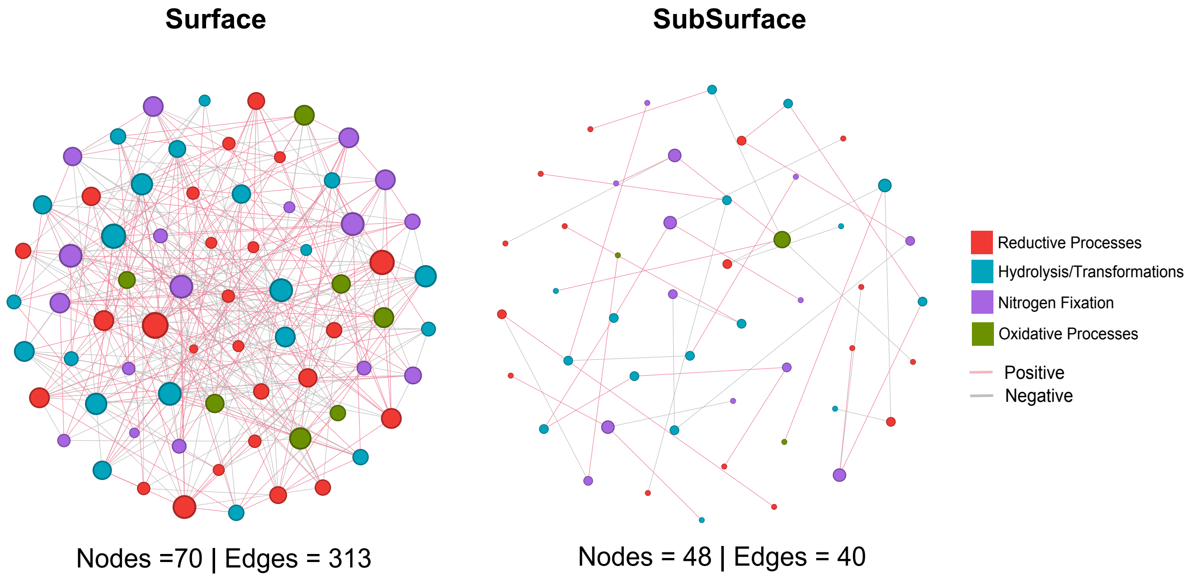

Figure S5. Co-occurrence networks of nitrogen-cycling genes in surface and subsurface peat samples inferred using the SparCC algorithm. Only statistically significant correlations are shown ($|r| \geq 0.3$, $p < 0.05$). Nodes represent individual N-cycling genes coloured by functional pathway. Edges represent positive (red) or negative (grey) correlations, and node size is proportional to degree centrality (number of connections). Network topology metrics indicate that surface networks were more complex and cohesive (70 nodes, 313 edges, network density = 0.13, average degree = 8.94) compared with subsurface networks (48 nodes, 40 edges, network density = 0.035, average degree = 1.67).

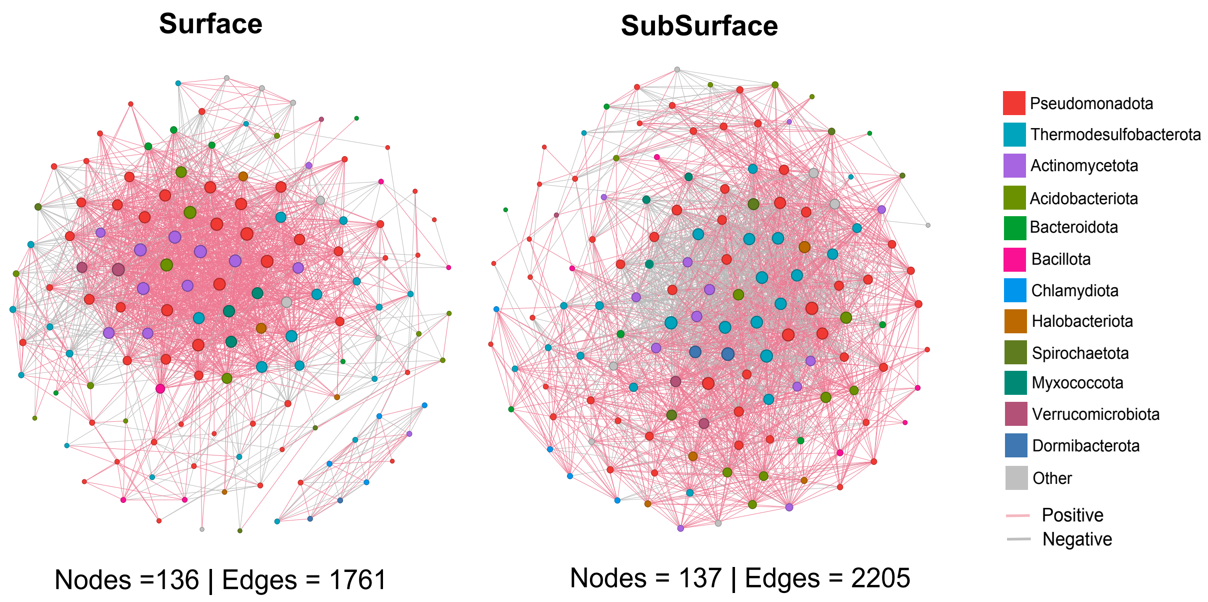

Figure S6. Taxonomic co-occurrence networks of metagenome-assembled genomes (MAGs) inferred using the SparCC algorithm from abundance correlations across samples. Networks include 140 high-quality MAGs (>75% completeness, <5% contamination). Only statistically significant correlations are shown ($|r| \geq 0.3$, $p < 0.05$). Nodes represent individual MAGs coloured by phylum, and node size is proportional to degree centrality (number of connections). Edges represent positive (pink) or negative (grey) correlations. Network topology metrics indicate that subsurface communities formed denser and more connected networks (137 nodes, 2205 edges, network density = 0.237, average degree = 32.19) than surface communities (136 nodes, 1761 edges, network density = 0.192, average degree = 25.9), suggesting stronger taxonomic connectivity in subsurface peat communities that may reflect increased metabolic interdependence under oligotrophic, anoxic conditions.

Supplementary Tables

Table S1. Physicochemical properties of peat samples corresponding to NCBI BioSample accession numbers SAMN50450969–SAMN50450986.

Sample	Depth	BioSample Accession	SRA	TN (%)	TC (%)	TS (%)	TP (%)	EC (mS cm⁻¹)	pH	Moisture (%)
GAT	0-20cm	SAMN50450972	SRS26073066	1.87	49.79	0.975	0.0953	0.19	3.69	89.770
GBT	0-20cm	SAMN50450973	SRS26073067	1.86	48.87	0.721	0.0985	0.2	3.84	89.530
GCT	0-20cm	SAMN50450974	SRS26073068	1.81	49.01	0.737	0.1217	0.26	3.68	88.646
CAT	0-20cm	SAMN50450969	SRS26073055	1.71	48.58	0.768	0.1148	0.13	3.71	90.282
CBT	0-20cm	SAMN50450970	SRS26073056	1.53	47.33	0.645	0.1131	0.16	3.84	91.080
CCT	0-20cm	SAMN50450971	SRS26073065	1.73	48.54	0.771	0.1303	0.18	3.81	89.918
MAT	0-20cm	SAMN50450975	SRS26073069	2.03	50.19	0.666	0.1428	0.215	3.85	89.433
MBT	0-20cm	SAMN50450976	SRS26073070	1.95	49.41	0.666	0.1282	0.2	3.88	89.990
MCT	0-20cm	SAMN50450977	SRS26073071	1.98	49.36	0.643	0.1257	0.2	3.86	89.295
GAB	20-40cm	SAMN50450981	SRS26073059	1.44	55.58	0.536	0.0725	0.13	4.06	90.966
GBB	20-40cm	SAMN50450982	SRS26073060	1.45	50.5	0.511	0.0794	0.15	3.9	90.261
GCB	20-40cm	SAMN50450983	SRS26073061	1.16	52.26	0.471	0.0648	0.14	3.96	90.763
CAB	20-40cm	SAMN50450978	SRS26073072	1.47	49.77	0.475	0.1073	0.12	3.85	90.316
CBB	20-40cm	SAMN50450979	SRS26073057	1.43	50.3	0.48	0.1017	0.15	4.07	91.604
CCB	20-40cm	SAMN50450980	SRS26073058	1.48	48.05	0.56	0.0862	0.11	4.03	90.695
MAB	20-40cm	SAMN50450984	SRS26073062	1.36	51.23	0.472	0.0691	0.15	4.03	90.411
MBB	20-40cm	SAMN50450985	SRS26073063	1.6	51.77	0.467	0.0682	0.16	4.06	90.370
MCB	20-40cm	SAMN50450986	SRS26073064	1.52	51.68	0.485	0.0824	0.15	3.96	91.551

Table S2. Comparison of physicochemical properties between surface (0–20 cm) and subsurface (20–40 cm) peat layers using Mann–Whitney tests.

Variable	Surface mean	Subsurface mean	*p*-value	Significant
TN (%)	1.83	1.43	0.00057	Yes
TC (%)	49.01	51.24	0.00807	Yes
TS (%)	0.73	0.50	0.00041	Yes
TP (%)	0.119	0.081	0.00148	Yes
EC (mS cm^{-1})	0.193	0.140	0.00330	Yes
pH	3.80	3.99	0.00090	Yes
Moisture (%)	89.77	90.77	0.00472	Yes

Table S3. Functional genes involved in nitrogen cycling processes

N Cycling Functional Categories	Genes
Nitrogen Fixation	*nifA, nifX, nifW, nifV, nifD, nifN, nifK, nifH, nifB, nifE, nifT, nifHD2, nifZ, anfG, vnfD, vnfH, vnfK*
Ammonia Oxidation	*amoA, amoB, amoC*
Hydroxylamine Oxidatioreductase	*hcp, hao*
Nitrite Oxidation	*nxrA, nxrB*
Nitroalkane Oxidation	*nao*
Nitrate Reduction	*napA, napB, napC, napD, napE, napG, napH, narB, narV, narW, nasA, nirS, nirK*
Nitrite Reduction to Ammonium	*nirA, nirB, nirD, nrfD, nrfC, nirfF, nrfA*
Nitric Oxide Reduction	*norB, norC, norD, nosZ*
Organic Nitrogen Transformation	*gdhA, glsA, glnA, asnB, gltD, gdh2, gltS, gltB, ureA, ureB, ureC, nmo, glt1, gudB, ansB, dadA, gcvT, amiF, cynS, aspA, metC*

Table S4. Spearman correlations between KEGG *amo* genes and NCycDB *pmo* genes

Comparison	Spearman ρ	p-value	n
pmoA (NCycDB) vs *amoA* (KEGG)	0.918	1.5×10^{-7}	18
pmoB (NCycDB) vs *amoB* (KEGG)	0.943	9.7×10^{-9}	18
pmoC (NCycDB) vs *amoC* (KEGG)	0.974	1.1×10^{-11}	18

Table S5. Spearman correlations between KEGG and NCycDB annotations for selected nitrogen cycling genes and functional ratios.

Comparison	Spearman ρ	p-value	n
amoA (NCycDB vs KEGG)	0.940	7.4×10^{-9}	18
norB (NCycDB vs KEGG)	0.692	1.5×10^{-3}	18
nosZ (NCycDB vs KEGG)	0.639	4.3×10^{-3}	18
ureC (NCycDB vs KEGG)	0.959	7.6×10^{-10}	18
amoA/nosZ (NCycDB vs KEGG)	0.936	1.2×10^{-8}	18
amoA/norB (NCycDB vs KEGG)	0.940	7.4×10^{-9}	18
amoA/ureC (NCycDB vs KEGG)	0.939	8.6×10^{-9}	18

Table S6. Pearson correlations between KEGG and NCycDB annotations for selected nitrogen cycling genes and functional ratios.

Comparison	Pearson r	p-value	n
amoA (NCycDB vs KEGG)	0.967	6.2×10^{-11}	18
norB (NCycDB vs KEGG)	0.612	6.9×10^{-3}	18
nosZ (NCycDB vs KEGG)	0.661	3.1×10^{-3}	18
ureC (NCycDB vs KEGG)	0.978	4.1×10^{-12}	18
amoA/*nosZ* (NCycDB vs KEGG)	0.953	5.7×10^{-10}	18
amoA/*norB* (NCycDB vs KEGG)	0.946	1.6×10^{-9}	18
amoA/*ureC* (NCycDB vs KEGG)	0.961	1.7×10^{-10}	18

Table S7. Details on the key functional genes related to P cycling studied in the present work

Pathway	Gene	Enzyme
Organic P mineralisation	*phoA*	Alkaline phosphatase A
	phoD	Alkaline phosphatase D
	phoX	Alkaline phosphatase X
	phoN	Acid phosphatase class A
	aphA	Acid phosphatase class B
	olpA	Acid phosphatase class C
	appA	4-phytase
	3-phytase	3-phytase
	phnG	C–P lyase multienzyme complex
	phnH	C–P lyase multienzyme complex
	phnI	C–P lyase multienzyme complex
	phnJ	C–P lyase multienzyme complex
	phnK	C–P lyase multienzyme complex
	phnL	C–P lyase multienzyme complex
	phnM	C–P lyase multienzyme complex
Inorganic P solubilisation	*ppa*	Inorganic pyrophosphatase
	ppx	Exopolyphosphatase
	ppk1	Polyphosphate kinase
	gcd	Quinoprotein glucose dehydrogenase
	pqqC	Pyrroloquinoline quinone synthase C
P-starvation response regulation	*phoB*	Phosphate regulon response regulator
	phoR	Phosphate regulon sensor histidine kinase
	phoU	PhoR/phoB inhibitor protein

Table S8. Pairwise Welch t-test comparisons of $\log_2$ surface-to-subsurface ratios of nitrogen cycling processes among vegetation types.

Process	Eriophorum	Calluna	Sphagnum	Eriophorum vs Calluna	Calluna vs Sphagnum	Eriophorum vs Sphagnum
Oxidative	1.154	1.444	1.536	0.762	0.668	0.933
N fixation	0.209	0.432	0.535	0.223	0.783	0.196
Assimilatory	0.283	0.352	0.407	0.330	0.261	0.081
N hydrolysis / transformation	0.022	0.095	0.247	0.601	0.958	0.445
Reductive	-0.247	-0.092	0.122	0.680	0.646	0.475
Dissimilatory	-1.978	-1.787	-1.844	0.640	0.188	0.091

Table S9. Relative abundance (%) of dominant microbial phyla carrying nitrogen-cycling genes across peat samples under different vegetation types and depths. Values represent the percentage contribution

of each phylum to the total classified nitrogen-cycling gene annotations in each sample. Only dominant phyla are shown, while all remaining phyla are grouped as "Others." Unclassified taxa were excluded prior to analysis and the remaining phyla were re-normalised to sum to 100% within each sample.

Sample codes indicate vegetation type, soil depth, and replicate. Vegetation types include Calluna (C), Eriophorum (G), and Sphagnum (M). Soil depth is indicated by A (surface) and B (subsurface). Replicates are labelled A-C. Thus, for example, CAT, CBT, and CCT represent Calluna surface replicates A-C, whereas CAB, CBB, and CCB represent Calluna subsurface replicates A-C. Similarly, GAT-GCT and MAT-MCT denote surface samples under Eriophorum and Sphagnum vegetation, respectively, while GAB-GCB and MAB-MCB represent the corresponding subsurface samples.

Phylum	CAT	CAB	CCT	GAT	GBT	GCT	MAT	MBT	MCT	CAB	CBB	CCB	GAB	GBB	GCB	MAB	MBB	MCB
p_Pseudomonadota	69.12	71.09	72.30	69.32	72.19	70.68	75.97	68.04	74.14	66.78	57.04	65.08	60.60	71.77	64.52	61.78	69.56	60.74
p_Acidobacteriota	5.62	5.62	5.46	5.63	5.38	5.30	5.34	5.87	3.92	11.68	21.74	15.43	17.43	12.55	16.55	17.42	13.46	17.59
p_Bacteroidota	0.31	0.13	0	0.22	0.38	0.04	0.19	0.21	0.17	3.95	3.87	2.63	2.12	2.17	1.55	3.92	2.33	4.28
p_Actinomycetota	14.19	13.63	9.92	14.90	12.91	10.36	7.28	12.88	7.32	3.29	1.12	3.93	1.26	2.61	2.56	3.19	1.82	1.52
p_Thermodesulfobacteriota	0.41	0.52	0.79	0.61	0.70	0.96	1.37	1.22	1.38	3.18	3.01	2.17	3.92	1.34	2.56	3.50	3.46	2.17
p_Planctomycetota	4.95	5.05	3.23	6.13	3.60	5.89	4.46	4.66	4.15	1.97	2.15	1.85	2.83	1.94	1.96	1.21	1.26	1.45
p_Verrucomicrobiota	0	0	0.13	0.06	0.11	0	0.15	0.13	0.06	1.43	1.80	1.71	2.04	0.90	1.25	2.17	1.32	1.71
p_Candidatus Bathyarchaeota	0.15	0.13	0.31	0.11	0	0.17	0.11	0.17	1.21	1.32	1.63	0.65	1.57	1.05	1.01	1.75	1.26	1.78
p_Euryarchaeota	1.28	0.65	3.41	0.17	1.24	2.26	1.60	2.47	4.26	1.10	1.55	1.57	1.10	0.67	1.43	0.78	1.32	1.12
p_Bacillota	0	0.09	0.22	0.06	0.05	0.50	0.08	0.76	0.35	0.44	2.66	0.32	2.04	2.76	2.92	0.60	1.70	3.62
Others	3.98	3.09	4.24	2.81	3.44	3.84	3.43	3.61	3.05	4.88	3.44	4.67	5.10	2.24	3.69	3.68	2.52	4.02

Table S10. Details on the key functional genes related to N cycling studied in the present work

Nitrogen cycling process	Gene	Function
Nitrogen hydrolysis / organic N transformation	*ureC*	Urease alpha subunit (urea → NH_3)
Nitrogen hydrolysis / organic N transformation	*glsA*	Glutaminase (glutamine → glutamate + NH_3)
Nitrogen hydrolysis / organic N transformation	*asnB*	Asparagine synthase
Nitrogen hydrolysis / organic N transformation	*ansB*	L-asparaginase (asparagine → aspartate + NH_3)
Nitrogen hydrolysis / organic N transformation	*amiF*	Amidase (amide hydrolysis releasing NH_3)
Nitrogen hydrolysis / organic N transformation	*cynS*	Cyanase (cyanate → NH_3 + CO_2)
Nitrogen hydrolysis / organic N transformation	*gdhA*	Glutamate dehydrogenase
Nitrogen hydrolysis / organic N transformation	*gudB*	Glutamate dehydrogenase
Nitrogen hydrolysis / organic N transformation	*nmo*	Nitronate monooxygenase
Nitrogen hydrolysis / organic N transformation	*gcvT*	Glycine cleavage system T protein
Nitrogen hydrolysis / organic N transformation	*aspA*	Aspartate ammonia-lyase
Nitrogen hydrolysis / organic N transformation	*dadA*	D-amino acid dehydrogenase
Nitrogen hydrolysis / organic N transformation	*metC*	Cystathionine β-lyase
Nitrogen fixation	*nifD*	Nitrogenase molybdenum-iron protein alpha chain
Nitrogen fixation	*nifK*	Nitrogenase molybdenum-iron protein beta chain
Nitrification	*amoA*	Ammonia monooxygenase subunit A
Nitrification	*amoB*	Ammonia monooxygenase subunit B
Nitrification	*amoC*	Ammonia monooxygenase subunit C
Hydroxylamine reductase	*hcp*	Hydroxylamine reductase
Nitrate reduction	*napA*	Periplasmic nitrate reductase catalytic subunit
Nitrate reduction	*napB*	Periplasmic nitrate reductase small subunit
Nitrate reduction	*narB*	Ferredoxin-nitrate reductase
Nitrite oxidation	*nxrA*	Nitrite oxidoreductase alpha subunit
Nitrite oxidation	*nxrB*	Nitrite oxidoreductase beta subunit
Assimilatory nitrate reduction	*nasA*	Assimilatory nitrate reductase
Dissimilatory nitrite reduction (DNRA)	*nrfA*	Cytochrome c nitrite reductase
Denitrification	*nirK*	Copper-containing nitrite reductase
Denitrification	*norB*	Nitric oxide reductase subunit B
Denitrification	*norC*	Nitric oxide reductase subunit C
Denitrification	*nosZ*	Nitrous oxide reductase
